# Mast cells support lung eosinophil homeostasis and the acute innate immune response to respiratory syncytial virus

**DOI:** 10.1038/s41467-026-73438-w

**Published:** 2026-05-24

**Authors:** Roopa Hebbandi Nanjundappa, Christopher R. Liwski, Alexander Edgar, Matthieu Castonguay, Ian D. Haidl, Jean S. Marshall

**Affiliations:** 1 Department of Microbiology and Immunology, Dalhousie University, Halifax, NS Canada; 2 Department of Pathology, Dalhousie University, Halifax, NS Canada; 3 Beatrice Hunter Cancer Research Institute, Halifax, NS Canada

**Keywords:** Mast cells, Viral infection, Eosinophils

## Abstract

As tissue-resident immune cells, mast cells release inflammatory agents in response to local viral infections. Here, we analyze *Cpa3-Cre*; *Mcl-1*^fl/fl^ mice, which lack mast cells, to study the consequences of mast cell deficiency during respiratory syncytial virus (RSV) infection. At the early stages of RSV infection, mast cell-deficient mice exhibit higher viral loads, greater weight loss and exacerbated lung tissue damage when compared to control mice. Mast cell deficiency also decreases eosinophil recruitment, while increasing the influx of inflammatory monocytes and the levels of CXCL10, CCL4, and TNF in lung tissue. Reconstitution of bone-marrow–derived mast cells into mast cell-deficient mice restores eosinophil responses and protection from RSV infection, as well as repletes lung eosinophil numbers and GM-CSF levels in non-RSV conditions. Our data thus demonstrate that mast cells support an effective antiviral response and lung protection early in RSV infection and also help maintain lung eosinophil homeostasis, thereby placing them as important regulators of antiviral defense.

## Abstract (web summary)

Respiratory syncytial virus (RSV) is a widespread pathogen, frequently causing bronchiolitis and pneumonia. Here, the authors analyze mast cell-deficient mice, showing that mast cells support the early antiviral response and limit lung damage during RSV infection.

## Introduction

Mast cells are tissue-resident immune cells with diverse roles in host defense and immune regulation^1,2^. They have been implicated in host defense against several parasites and bacteria^3–5^. In addition to granule-associated mediators such as histamine, proteoglycans, and proteases that are released upon activation, mast cells can also produce multiple lipid mediators, cytokines, and chemokines. Often situated near blood vessels, mast cells regulate vascular permeability and recruit immune cells, including neutrophils and eosinophils, to sites of infection^6^. In the airways, their strategic localization at epithelial surfaces, around blood vessels, and the impacts of their mediators on smooth muscle enables them to modulate tissue remodeling, inflammation, and bronchoconstriction^7,8^.

Respiratory syncytial virus (RSV) is a major cause of lower respiratory tract infections, including bronchiolitis and pneumonia, particularly affecting young children, the elderly, and immunocompromised populations^9,10^. RSV infection is responsible for more than 33 million cases of lower respiratory tract infections annually and has been suggested to predispose individuals to long-term respiratory problems, including asthma^11^. Vaccines and prophylactic antibodies are not available in many countries, underscoring the need for a better understanding of host immune responses to RSV to inform prevention and treatment strategies.

While human mast cells are relatively resistant to productive RSV infection in vitro, they produce type I interferons (IFN-α2, IFN-β) and chemokines (CCL3, CCL4, and CXCL10) in response to the virus^12,13^. Human mast cells contribute to antiviral immunity by secreting chemokines such as CXCL8, CCL3, and CCL5, which recruit natural killer (NK) cells and CD56^+^ T cells to the sites of infection^14,15^. They also facilitate endothelial activation through IL-1β and TNF, thereby promoting the local recruitment of effector immune cells during viral infections^16^. Their involvement has been documented in multiple settings including coronavirus, human immunodeficiency virus (HIV), and dengue virus infections, where they can exacerbate inflammation, contribute to tissue damage, or worsen disease severity^17–20^. While RSV has been extensively studied in relation to wheezing and asthma-associated inflammation^21–23^, conditions in which mast cells are well-established contributors^24,25^, the specific role of mast cells in early antiviral defense and RSV-associated lung pathology remains poorly defined.

In this study, we use the mast cell deficient *Cpa3-Cre; Mcl-1*^*fl/fl*^ mouse model, which selectively lacks mast cells, to investigate the role of mast cells during RSV infection. Mast cell-deficient mice exhibit higher viral loads, greater weight loss, and increased lung inflammatory tissue damage during early RSV infection compared to mast cell-containing wild-type (WT) controls. Their lungs also exhibit elevated levels of inflammatory cytokines and chemokines (e.g., CCL4, CXCL10, TNF) and an increased influx of inflammatory monocytes. At baseline, mast cell-containing mice exhibit higher lung eosinophil frequencies, indicating a role for mast cells in maintaining tissue eosinophil homeostasis. Reconstitution of mast cells in *Cpa3-Cre; Mcl-1*^*fl/fl*^ mice restores control of viral infection and reduces inflammatory pathology. These findings identify mast cells as important regulators of early antiviral defense and immune homeostasis in the lung during RSV infection.

## Results

### The presence of mast cells is associated with reduced weight loss, viral load, and pulmonary tissue damage

**Fig. 1:**
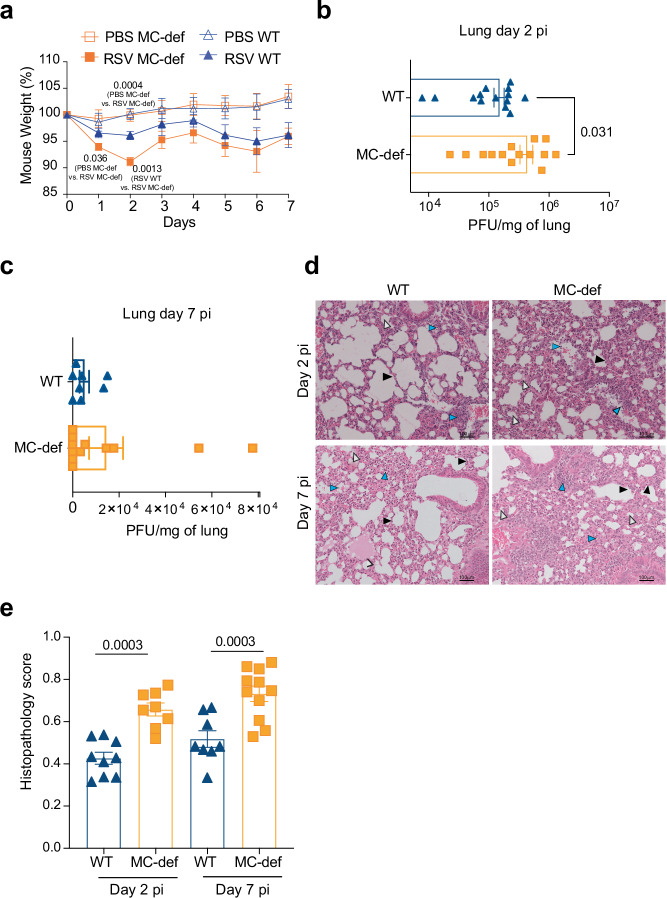
Mast cells limit early weight loss, reduce viral burden, and protect against pulmonary tissue damage during RSV infection. Mast cell-sufficient wild-type (WT) and mast cell-deficient *Cpa3-Cre; Mcl-1*^*fl/fl*^ (MC-def) mice were infected intranasally with 4 × 10^6^ PFU RSV and assessed at days 2 and 7 postinfection (pi). **a**. Body weight changes over time in RSV- or mock-infected (PBS) WT (*n* = 9) and MC-def (*n* = 5) mice. *P* values for comparisons between RSV-infected WT (*n* = 22) and RSV-infected MC-def (*n* = 24) mice, and between mock-infected PBS-administered and RSV-infected mast cell-deficient mice, are indicated in the graph. Data were compiled from three experiments. Statistical significance was assessed using Kruskal–Wallis test with Dunn’s multiple comparison. **b**. RSV viral loads in lung tissue on day 2 pi of WT and mast cell-deficient mice. WT (*n* = 14) versus MC-def (*n* = 14) mice on day 2 pi, data were compiled from four experiments. Statistical significance was assessed by a two-sided Mann–Whitney *U* test. **c**. RSV viral loads in lung tissue at day 7 pi of WT and mast cell-deficient mice. WT (*n* = 8) versus MC-def (*n* = 12) mice on day 7 pi, data were compiled from two experiments. Statistical significance was assessed by a two-sided Mann–Whitney *U* test. **d**. Representative lung histology images from RSV-infected WT and MC-def mice at day 2 pi (upper panels) and day 7 pi (lower panels), showing monocytic inflammation in alveolar and interstitial spaces (blue arrows), hyaline membranes and proteinaceous debris within airspaces (white arrows), and alveolar septal thickening (black arrows). **e**. Lung histopathology scores assessed using the ASL/ALI scoring system. 2 days pi: WT (*n* = 9), MC-def (*n* = 8) mice; 7 days pi: WT (*n* = 8), MC-def (*n* = 11) mice. Data were compiled from two experiments. Statistical significance was assessed using one-way ANOVA with Holm–Sidak multiple comparison. Data are presented as mean ± SEM in all graphs. Exact *P* values are indicated in each graph. Source data are provided as a Source data file.

To investigate the role of mast cells in the response to RSV infection, mast cell-deficient *Cpa3-Cre; Mcl-1*^*fl/fl*^ mice^26^ and their age-matched WT controls were infected with RSV, and body weight was monitored over time following infection. Body weight loss was significantly greater in mast cell-deficient mice than WT mice on days 1 and 2 postinfection (pi) (Fig. 1a) but not at the later time points. Significantly higher viral loads were observed in the lungs of RSV-infected *Cpa3-Cre; Mcl-1*^*fl/fl*^ mice compared to WT mice when assessed by ddPCR at day 2 pi (Fig. 1b), but not day 7 pi (Fig. 1c).

Lung tissues from RSV-infected mice were examined for inflammatory pathology using the validated ASL/ALI scoring system^27,28^. On both day 2 and day 7 pi, most mice exhibited monocytic inflammation in the alveolar and interstitial spaces, the presence of hyaline membranes, proteinaceous debris within the airspaces, and alveolar septal thickening (Fig. 1d). RSV-infected mast cell-deficient *Cpa3-Cre; Mcl-1*^*fl/fl*^ mice demonstrated significantly more severe lung pathology scores when compared with WT mice (Fig. 1e), suggesting that mast cells play a role in limiting both the level and impact of RSV infection in vivo.

### Mast cells regulate inflammatory monocytes and eosinophil infiltration

**Fig. 2:**
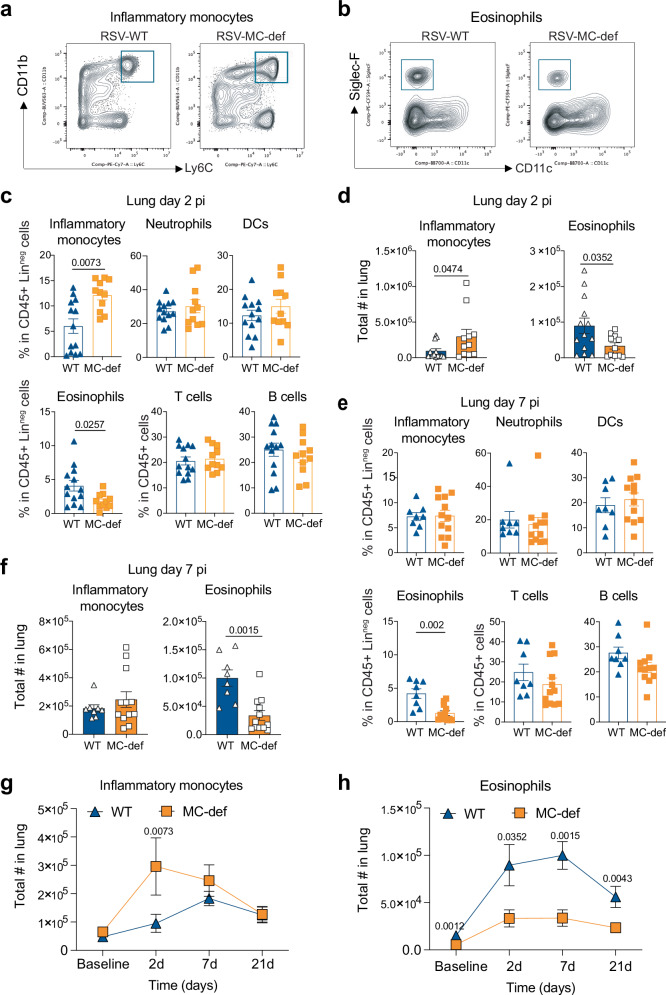
Mast cells regulate inflammatory monocyte infiltration and promote eosinophil responses during RSV infection. Mast cell-sufficient wild-type (WT) and mast cell-deficient *Cpa3-Cre; Mcl-1*^*fl/fl*^ (MC-def) mice were intranasally infected with 4 × 10^6^ PFU RSV and analyzed for lung immune cell infiltration by flow cytometry at various postinfection (pi) time points using the gating strategy shown in Supplementary Fig. 1a. **a, b**. Representative contour plots showing inflammatory monocytes (CD11b^high^Ly6C^+^) (**a**) and eosinophils (Siglec-F^+^CD11c^−^) (**b**) in lung single-cell suspensions from RSV-infected WT and MC-def mice. **c**. Percentage of inflammatory monocytes, neutrophils, DCs, eosinophils, T and B cells within CD45^+^ lineage-negative (Lin^neg^: CD3^−^CD19^−^), or CD45^+^ cells in the lung tissues of WT (*n* = 13) versus MC-def (*n* = 11) mice infected with RSV at day 2 pi. Data were compiled from three experiments. **d**. Total numbers of inflammatory monocytes and eosinophils in the lung tissues of WT and MC-def mice depicted in panel **c**. Data were compiled from three experiments. **e**. Percentage of inflammatory monocytes, neutrophils, DCs, eosinophils, T and B cells within CD45^+^ lineage-negative (Lin^neg^: CD3^−^CD19^−^), or CD45^+^ cells in the lung tissues of WT (*n* = 8) versus MC-def (*n *= 12) mice infected with RSV at day 7 pi. Data were compiled from two experiments. **f**. Total numbers of inflammatory monocytes and eosinophils in the lung tissues of WT and MC-def mice depicted in panel **e**. Data were compiled from two experiments. **g**, **h**. Kinetics of total counts of inflammatory monocytes (**g**) and eosinophils (**h**) in lung tissues of WT and MC-def mice at baseline and days 2, 7, and 21 pi. Baseline: WT (*n* = 7), MC-def (*n* = 10); 2 days pi: WT (*n* = 13), MC-def (*n* = 11); 7 days pi: WT (*n* = 8), MC-def (*n* = 12); 21 days pi: WT (*n* = 6), MC-def (*n* = 6). Statistical significance was assessed using two-sided Mann–Whitney *U* tests. Data are presented as mean ± SEM in all the graphs. Exact significant *P* values are indicated in each graph. Source data are provided as a Source data file.

To understand the potential mechanisms behind the heightened inflammation observed in the absence of mast cells, we analyzed immune cell infiltration and inflammatory mediators in RSV-infected mast cell-deficient *Cpa3-Cre; Mcl-1*^*fl/fl*^ mice compared with RSV-infected WT mice. Immune cell infiltration analysis using the gating strategy as shown (Supplementary Fig. 1a) at day 2 pi revealed that the lungs of RSV-infected *Cpa3-Cre; Mcl-1*^*fl/fl*^ mice had increased inflammatory monocyte infiltration (CD11b^+^Ly6C^high^) but significantly lower eosinophils than WT mice (Fig. 2a–d).

No significant difference was observed in the percentage of inflammatory monocytes in the lung tissue between mast cell-deficient *Cpa3-Cre; Mcl-1*^*fl/fl*^ and WT mice at days 7 (Fig. 2e, f) and 21 pi (Fig. 2g). However, eosinophil numbers remained significantly lower in the lungs of *Cpa3-Cre; Mcl-1*^*fl/fl*^ mice (Fig. 2e, f) at these time points (Fig. 2h). RSV-infected WT mice exhibited increased eosinophil density in the lungs on days 2, 7, and 21 pi compared with RSV-infected *Cpa3-Cre; Mcl-1*^*fl/fl*^ mice (Fig. 2h). These cells are known to be retained in lung tissue for more than 15 days following viral infections^29^. The role of mast cells in regulating eosinophil responses appeared highly selective since no differences were observed in the percentages and overall lung tissue numbers of total T cells, B cells, neutrophils, dendritic cells (DC), NK cells, or CD8^+^ T cells between RSV-infected WT and mast cell-deficient mice at days 2 and 7 pi (Fig. 2c, e and Supplementary Fig. 2a, b).

To assess whether RSV infection alters mast cell abundance in lung tissue, we quantified granulated mast cells in the trachea and lung parenchyma. Histological analyses showed no change in mast cell density in the trachea or lung parenchyma (Supplementary Fig. 2c, d). These data indicate that RSV infection does not deplete lung mast cells.

### Mast cells regulate pulmonary inflammatory mediators

**Fig. 3:**
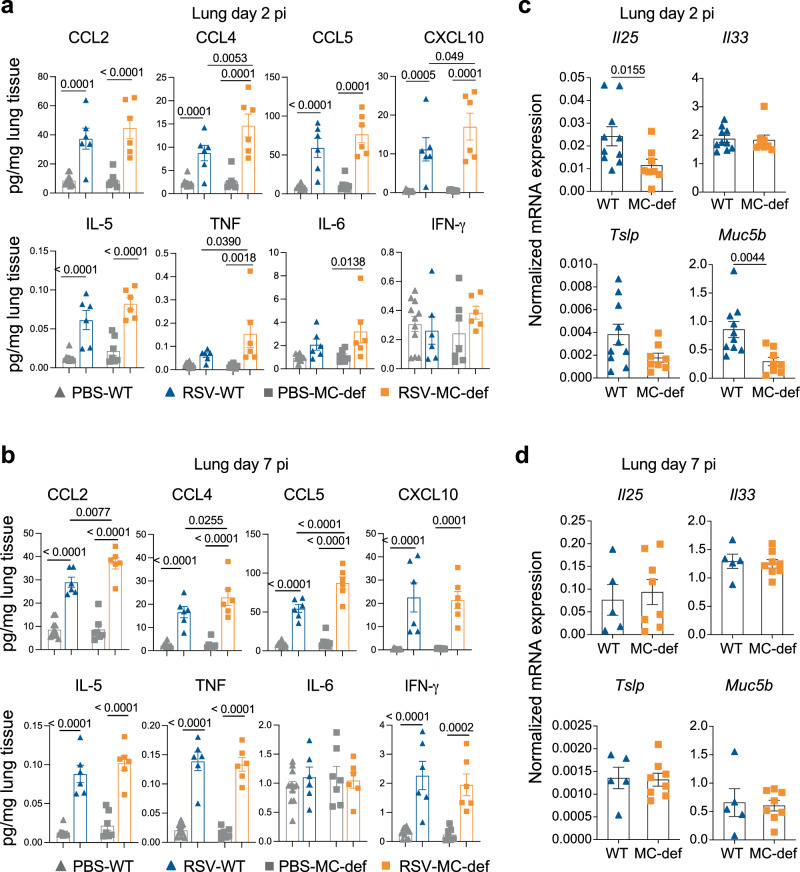
Mast cells regulate local lung cytokine responses, mucins, and IL-25 during RSV infection. Mast cell-sufficient wild-type (WT) and mast cell-deficient *Cpa3-Cre; Mcl-1*^*fl/fl*^ (MC-def) mice were intranasally infected with 4 × 10^6^ PFU RSV and assessed at various postinfection (pi) time points. **a**, **b**. Profiling of cytokines and chemokines in lung tissues of WT versus MC-def mice infected with RSV or treated with PBS on day 2 pi (**a**) and on day 7 pi (**b**). PBS-WT (*n* = 11), RSV-WT (*n* = 6), PBS-MC-def (*n* = 7), RSV-MC-def (*n* = 6) at each time point. Data were compiled from two experiments and were analyzed by one-way ANOVA with Holm–Sidak multiple comparisons test. **c**, **d**. Lung gene expression analysis on day 2 pi (**c**) and on day 7 pi (**d**) for *Il25, Il33, Tslp,* and *Muc5b*. Day 2 pi: WT (*n* = 10), MC-def (*n* = 8); day 7 pi: WT (*n* = 5), MC-def (*n* = 8). Data were compiled from two experiments and were analyzed by two-sided Mann–Whitney *U* test. Data are presented as mean ± SEM in all the graphs. Exact significant *P* values are indicated in each graph. Source data are provided as a Source data file.

Cytokine analyses of lung tissue indicated elevated levels of CCL4, CXCL10, IL-6, and TNF in RSV-infected mast cell-deficient *Cpa3-Cre; Mcl-1*^*fl/fl*^ mice compared with similarly infected WT mice at day 2 pi (Fig. 3a), in keeping with the increased viral load (Fig. 1b). There are also large numbers of inflammatory monocytes observed in the lungs of infected *Cpa3-Cre; Mcl-1*^*fl/fl*^ mice (Fig. 2c, d), which are known to produce substantial amount of CCL4, IL-6, and TNF^30,31^. Hence, there were increased levels of CCL4, CXCL10, IL-6, and TNF in lung tissues of RSV-infected *Cpa3-Cre; Mcl-1*^*fl/fl*^ mice compared to WT mice (Fig. 3a). However, there were no significant differences in lung tissue levels of IL-1β, IL-10, IL-33, IL-4, IL-13, or IFN-γ between RSV-infected WT and *Cpa3-Cre; Mcl-1*^*fl/fl*^ mice (Fig. 3a and Supplementary Fig. 2f).

At day 7 pi, cytokine analyses showed that the levels of CCL2, CCL4, and CCL5 in lung tissues were significantly higher in mast cell-deficient *Cpa3-Cre; Mcl-1*^*fl/fl*^ mice compared to WT mice, suggesting prolonged inflammation in the absence of mast cells (Fig. 3b), that correlated with the increased pulmonary damage (Fig. 1d, e). Other cytokines, including IL-1β, IL-10, IL-33, IL-4, and IL-13, remained unchanged at this time point (Supplementary Fig. 2g). No significant differences were detected in serum cytokines at day 7 pi in either WT or *Cpa3-Cre; Mcl-1*^*fl/fl*^ mice infected with RSV compared to PBS-treated mice (Supplementary Fig. 3a), suggesting that mast cell-dependent responses were largely localised to lung tissue.

IL-25 promotes the recruitment and accumulation of eosinophils at sites of inflammation^32–34^. Notably, *Il25* mRNA levels were elevated in RSV-infected WT mice compared to *Cpa3-Cre; Mcl-1*^*fl/fl*^ mice at day 2 pi (Fig. 3c), but not day 7 pi (Fig. 3d), correlating with enhanced eosinophil responses (Fig. 2c, d). By contrast, expression of *Il33* and *Tslp* did not differ between RSV-infected WT and *Cpa3-Cre; Mcl-1*^*fl/fl*^ mice at day 2 and 7 pi (Fig. 3c). Mucins are major glycoprotein components of the respiratory tract and play distinct roles in host defense. Muc5b has been implicated in protection against infections^35,36^. RSV-infected mast cell-containing mice, which exhibited a lower viral burden at day 2 pi (Fig. 1b), also showed increased *Muc5b* expression at day 2 pi, but not day 7 pi, relative to RSV-infected *Cpa3-Cre; Mcl-1*^*fl/fl*^ mice (Fig. 3c, d), supporting the concept that alterations in mucosal secretions could contribute to RSV clearance. Expression of *Il25, Il33*, *Tslp,* and *Muc5b* did not change at baseline between WT versus *Cpa3-Cre; Mcl-1*^*fl/fl*^ mice (Supplementary Fig. 2e). The expression of *Muc5ac* remained below the limit of detection in all groups of mice.

### Mast cells respond to RSV infection and their soluble mediators promote antiviral responses in airway epithelial cells

**Fig. 4:**
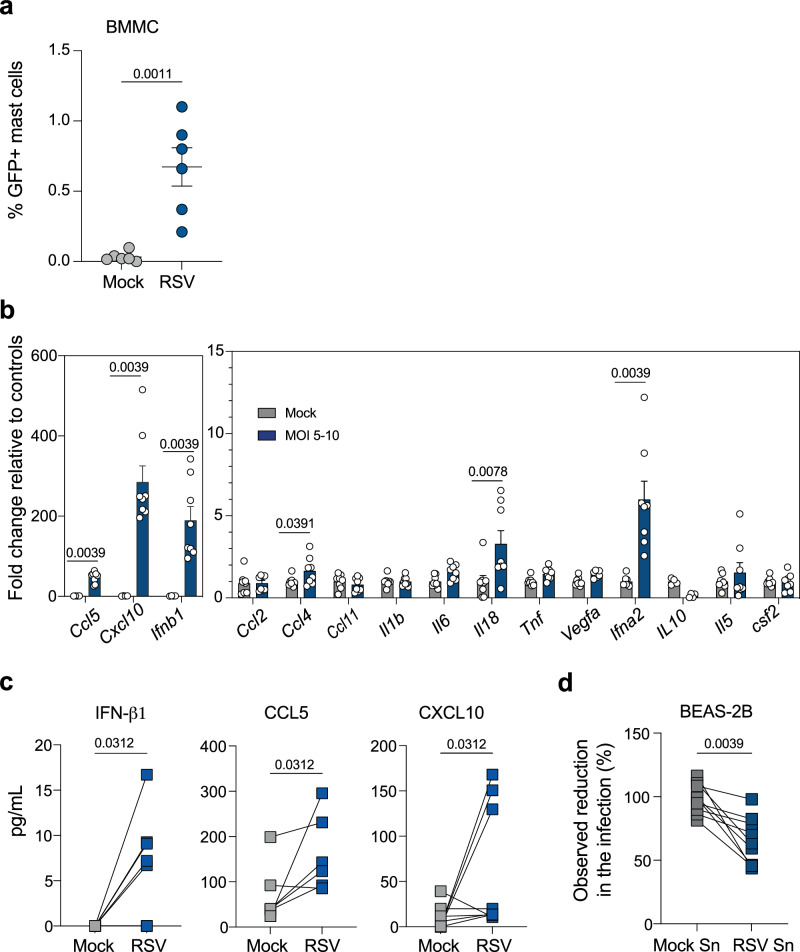
Bone marrow-derived mast cell responses to RSV infection. In vitro-cultured bone marrow-derived mast cells (BMMCs) were infected with RSV and examined for expression of GFP, production of cytokines, chemokines, and IFNs. **a**. Percentage of GFP^+^ cells in BMMCs infected with RSV or mock-treated (*n* = 6 per condition). Data were compiled from two experiments and were analyzed by a one-sided Mann–Whitney *U* test. **b**. Fold-change in gene expression of BMMCs infected with RSV compared to mock-treated controls (*n* = 8). Data were compiled from two experiments and were analyzed by a one-sided Wilcoxon matched-pairs signed-rank test. **c**. Protein levels of CCL5 (*n* = 6), CXCL10 (*n* = 8), and IFN-β1 (*n* = 8) in BMMC supernatants at 24 h postinfection (pi). Data were compiled from two experiments and were analyzed by a one-sided Wilcoxon matched-pairs signed-rank test. **d**. BEAS-2B airway epithelial cells were infected with GFP-expressing RSV in the presence of supernatants (Sn) from mock-treated or RSV-infected human cord blood-derived mast cells (CBMCs). RSV infection was quantified as the percentage of GFP^+^ epithelial cells and normalized to mock-treated controls (*n* = 9). Data were compiled from two experiments, and statistical analysis was performed using a two-sided Wilcoxon matched-pairs signed-rank test. Data are presented as mean ± SEM in all the graphs. Exact significant *P* values are indicated in each graph. Source data are provided as a Source data file.

Treatment of murine cultured bone marrow-derived mast cells (BMMCs) in vitro with RSV provided limited evidence of productive RSV infection, with only about 0.5% of BMMCs exhibiting GFP expression (Fig. 4a), similar to their human counterparts^12,13^. However, BMMCs exhibited robust mediator responses to RSV exposure, including the expression of CCL5, CXCL10, and IFNs at both the mRNA (Fig. 4b) and protein levels 24 h pi (Fig. 4c), suggesting that murine mast cells respond similarly to their human counterparts when exposed to RSV^12,13^. These findings confirm that mast cells are an important source of type I IFNs and selected chemokines, which would allow them to contribute effectively to protect against RSV infection.

To determine whether mast cell-derived mediators exert a direct antiviral effect on RSV, we exposed BEAS-2B airway epithelial cells to supernatants from either mock-treated or RSV-infected cord blood-derived mast cells (CBMCs) during viral infection. Quantification of GFP^+^ RSV-infected epithelial cells revealed that supernatants from RSV-infected CBMCs markedly reduced the numbers of infected BEAS-2B cells, compared with mock mast cell supernatant-treated controls, (Fig. 4d). These data demonstrated that mast cells release soluble antiviral factors upon RSV infection that can directly limit subsequent epithelial cell infection. Together with cytokine profiling, these findings support that mast cells contribute to early antiviral defense through secretion of antiviral mediators, including type I IFNs.

To determine whether mast cells modulate type I IFN responses in vivo during RSV infection, we quantified IFN gene expression in RSV-infected WT and mast cell-deficient *Cpa3-Cre; Mcl-1*^*fl/fl*^ lungs at day 2 pi. RSV infection induced comparable expression of *Ifnb1* and multiple *Ifna* subtypes in WT and *Cpa3-Cre; Mcl-1*^*fl/fl*^ mice (Supplementary Fig. 3b). *Ifnb1* expression levels positively correlated with viral load (Supplementary Fig. 3c), consistent with increased viral replication in *Cpa3-Cre; Mcl-1*^*fl/fl*^ mice rather than impaired IFN induction. No significant differences were observed in the expression of *Ifna1*, *Ifna2*, *Ifna14*, *Ifnl2*, or *Ifnl3* between mast cell-containing and mast cell-deficient animals.

### Mast cells modulate viral load, monocyte recruitment, and eosinophil dynamics

**Fig. 5:**
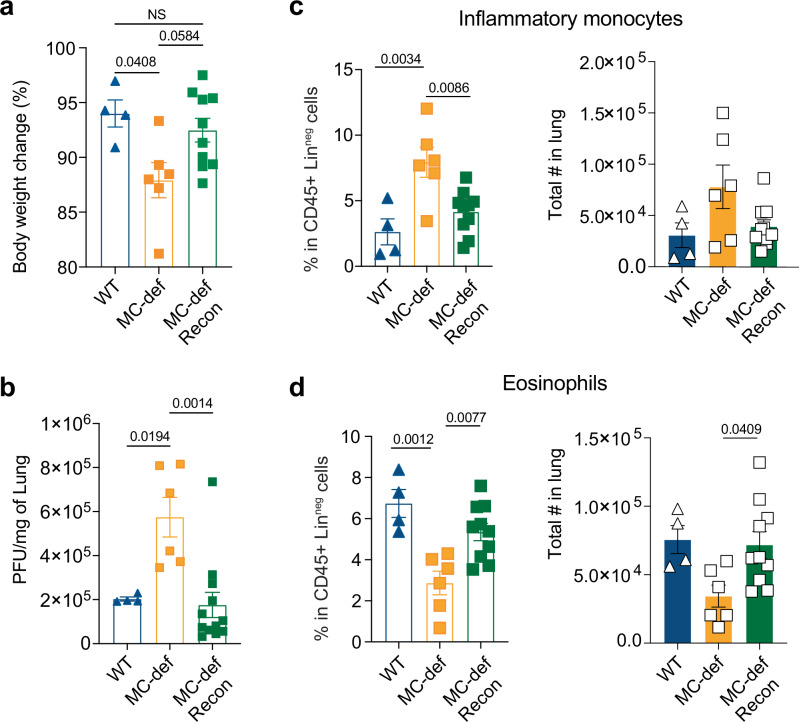
Mast cells reduce viral load and inflammatory monocytes in reconstituted mice. Mast cell-containing wild-type (WT), mast cell-deficient *Cpa3-Cre; Mcl-1*^*fl/fl*^ (MC-def), and mast cell-reconstituted *Cpa3-Cre; Mcl-1*^*fl/fl*^ (MC-def-Recon) mice were infected intranasally with 4 × 10^6^ PFU RSV and analyzed at day 2 postinfection (pi). **a**, **b**. Weight loss relative to initial body weight (**a**) and quantification of RSV load in the lung tissues (**b**) of WT (*n* = 4), MC-def (*n* = 6), and MC-def mice reconstituted with bone marrow-derived mast cells (BMMCs) (MC-def-Recon, *n* = 10) mice on day 2 pi. Data were compiled from two experiments and were analyzed by one-way ANOVA with Sidak’s multiple comparisons test. **c**, **d**. Percentage and total cell number of inflammatory monocytes (**c**) and eosinophils (**d**) within CD45^+^Lin^neg^ in the lung tissue of WT (*n* = 4), MC-def (*n* = 6), and MC-def reconstituted with BMMC (MC-def-Recon, *n* = 10) mice infected with RSV on day 2 pi. Data were compiled from two experiments and were analyzed by one-way ANOVA with Sidak’s multiple comparisons test. Data are presented as mean ± SEM in all the graphs. Exact significant *P* values are displayed in each graph. Source data are provided as a Source data file.

To further assess whether the contribution of mast cells to the reduced viral load and altered immune responses in RSV infected mice, we reconstituted mast cell-deficient *Cpa3-Cre; Mcl-1*^*fl/fl*^ mice with BMMCs i.v. and allowed mast cells to localize and mature in situ. Age-matched mast cell-deficient, mast cell-containing, and mast cell-reconstituted mice were then infected with RSV and assessed on day 2 pi. Mast cell-reconstituted *Cpa3-Cre; Mcl-1*^*fl/fl*^ mice exhibited significantly reduced weight loss (Fig. 5a) and lower viral loads in the lungs (Fig. 5b) compared with unreconstituted *Cpa3-Cre; Mcl-1*^*fl/fl*^ mice. Moreover, reconstitution with mast cells reduced the frequency of inflammatory monocytes and restored normal eosinophil recruitment to the lungs (Fig. 5c, d). Importantly, no significant differences were observed in weight loss or viral load between WT mice and mast cell-reconstituted *Cpa3-Cre; Mcl-1*^*fl/fl*^ mice (Fig. 5a, b). These findings confirmed that mast cells were critical for controlling the initial RSV viral load and limiting excessive inflammation.

### Mast cells selectively regulate eosinophils in the lung even in the absence of infection

Given the relationship between eosinophil responses and effective early antiviral immunity, we examined the mechanisms by which mast cells regulate eosinophil population in the lung. Mast cells are known to orchestrate eosinophil recruitment through mediator production and can produce cytokines that modify eosinophil survival and activation^37,38^. Inflammatory conditions are known to promote eosinophil activation, which is typically accompanied by increased expression of surface markers such as programmed death-ligand 1(PD-L1) and CD80^39^.

**Fig. 6:**
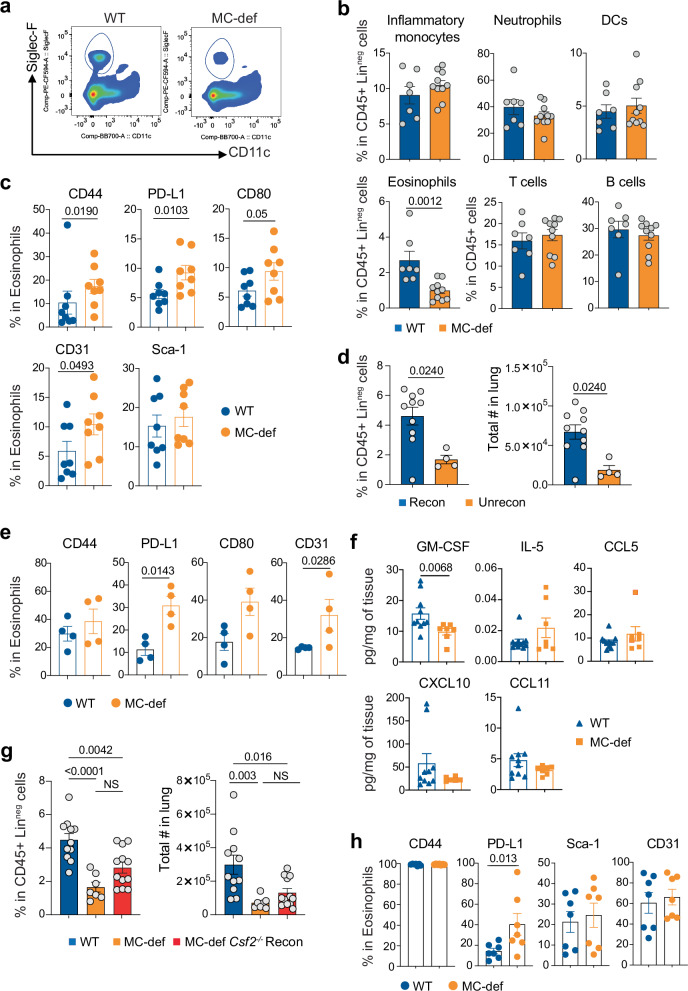
Mast cells regulate lung eosinophil numbers in the absence of infection. Uninfected mast cell-containing wild-type (WT) and mast cell-deficient *Cpa3-Cre; Mcl-1*^*fl/fl*^ (MC-def) mice were examined at 8–10 weeks of age. **a**. Representative flow cytometry plot illustrating eosinophil populations in lung tissue of WT and MC-def mice corresponding to the data in panel **b**. **b**. Percentage of immune cell subsets including inflammatory monocytes, neutrophils, DCs, eosinophils, T and B cells within CD45^+^Lin^neg^ or CD45^+^ populations in lung tissue of WT (*n* = 7) and MC-def (*n* = 10) mice. Data were compiled from two experiments and analyzed using a two-sided Mann–Whitney *U* test. **c**. Percentage of CD44-, PD-L1-, CD80-, CD31-, and Sca-1-expressing eosinophils in lung tissue of WT (*n* = 8) and MC-def (*n* = 8) mice. Data were compiled from two experiments and analyzed using a one-sided Mann–Whitney *U* test. **d**. Percentage within Lin^neg^ cells (left panel) and total number (right panel) of eosinophils in lung tissue of MC-def mice reconstituted with bone marrow-derived mast cells (BMMCs) (Recon, *n *= 10) versus MC-def mice (Unrecon, *n* = 4). Data were compiled from two experiments and analyzed using a two-sided Mann–Whitney *U*test. **e**. Percentage of CD44-, PD-L1-, CD80-, and CD31-expressing eosinophils in lung tissue of BMMC-reconstituted MC-def (Recon, *n* = 4) and non-reconstituted MC-def mice (Unrecon, *n* = 4). Data were obtained from one experiment and analyzed using a one-sided Mann–Whitney *U* test. **f**. Quantification of eosinophil chemoattractants GM-CSF, IL-5, CCL5, CXCL10, and CCL11 measured by ELISA in lung tissue of WT (*n* = 10) and MC-def (*n* = 7) mice. Data were compiled from two experiments and analyzed using a two-sided Mann–Whitney *U* test. **g**. Percentage within Lin^neg^ cells (left) and total number (right) of eosinophils in lung tissue of MC-def mice reconstituted with *Csf2*^−/−^ BMMC (MC-def *Csf2*^−/−^ Recon, *n* = 12), MC-def (*n* = 7), and WT (*n *= 11) mice. Data were compiled from two experiments and analyzed using one-way ANOVA with Holm–Sidak’s multiple comparisons test. **h**. Percentage of CD44-, PD-L1-, CD31-, and Sca-1-expressing eosinophils in lung tissue of WT and MC-def mice at day 2 pi (*n* = 7 per group). Data were compiled from two experiments and analyzed using a one-sided Mann–Whitney *U* test. Data are presented as mean ± SEM. Exact *P* values are indicated in each graph. NS indicates nonsignificant values (*P* > 0.05).

Uninfected mast cell-deficient mice displayed a highly selective decrease in lung eosinophil numbers and percentages, compared to WT mice, but no such disparity was evident in other immune cell populations (Fig. 6a, b and Supplementary Fig. 4a). Importantly, this reduction was selective to lung tissue, as no such differences in eosinophil levels were observed in the spleen, peritoneum, or blood (Supplementary Fig. 4b–d). Notably, eosinophils in mast cell-deficient *Cpa3-Cre; Mcl-1*^*fl/fl*^ mice exhibited elevated expression of activation markers, such as PD-L1, CD80, and CD31, in both lung (Fig. 6c) and spleen (Supplementary Fig. 4e), suggesting that mast cells influence eosinophil activation and recruitment even under homeostatic conditions. Reconstitution of *Cpa3-Cre; Mcl-1*^*fl/fl*^ mice with WT BMMCs restored normal eosinophil levels in the lung (Fig. 6d) and the eosinophils in such reconstituted animals displayed comparable activation marker expression, such as PD-L1, CD80 and CD31 (Fig. 6e) with WT mice. These observations confirmed that mast cell deficiency, directly or indirectly, affects eosinophil dynamics.

Multiple mast cell modulators can induce eosinophil chemotaxis, activation, and degranulation^37,38^. Cytokine and gene expression analyses of lung tissues for eosinophil chemoattractants and survival factors showed that uninfected WT mice had a selective increase in GM-CSF expression compared to mast cell-deficient *Cpa3-Cre; Mcl-1*^*fl/fl*^ mice (Fig. 6f and Supplementary Fig. 4f). GM-CSF has previously shown to contribute to the recruitment and retention of eosinophils in the mouse airways^40^. Reconstitution of *Cpa3-Cre; Mcl-1*^*fl/fl*^ mice with *Csf2*^−/−^ BMMC, in contrast to WT BMMC, did not restore normal eosinophil levels in *Cpa3-Cre; Mcl-1*^*fl/fl*^ mice. Notably, mice reconstituted with *Csf2*^−/−^ BMMC exhibited eosinophil populations similar to those observed in *Cpa3-Cre; Mcl-1*^*fl/fl*^ mice (Fig. 6g), suggesting a role for GM-CSF in driving or maintaining the number of pulmonary eosinophils in WT mice at baseline. Analysis of RSV-infected lung eosinophils in *Cpa3-Cre; Mcl-1*^*fl/fl*^ and WT mice revealed an increase in eosinophil activation markers in both *Cpa3-Cre; Mcl-1*^*fl/fl*^ and WT mice, although the number of eosinophils expressing PD-L1 in *Cpa3-Cre; Mcl-1*^*fl/fl*^ mice remained significantly higher than in WT (Fig. 6h).

Innate lymphoid cells type 2 (ILC2) localize in mucosal surfaces such as the lung and interact with mast cells during homeostasis and inflammation^41,42^. However, ILC2 percentages and counts, determined using the gating strategy shown in Supplementary Fig. 1b, did not differ between uninfected WT and *Cpa3-Cre; Mcl-1*^*fl/fl*^ mice in the lung or spleen (Supplementary Fig. 4g, h). These data suggest that, in contrast with eosinophils, in the absence of infection, resident mast cells do not regulate ILC2 numbers within lung tissue.

### Reduced eosinophil infiltration is not associated with increased monocyte infiltration

**Fig. 7:**
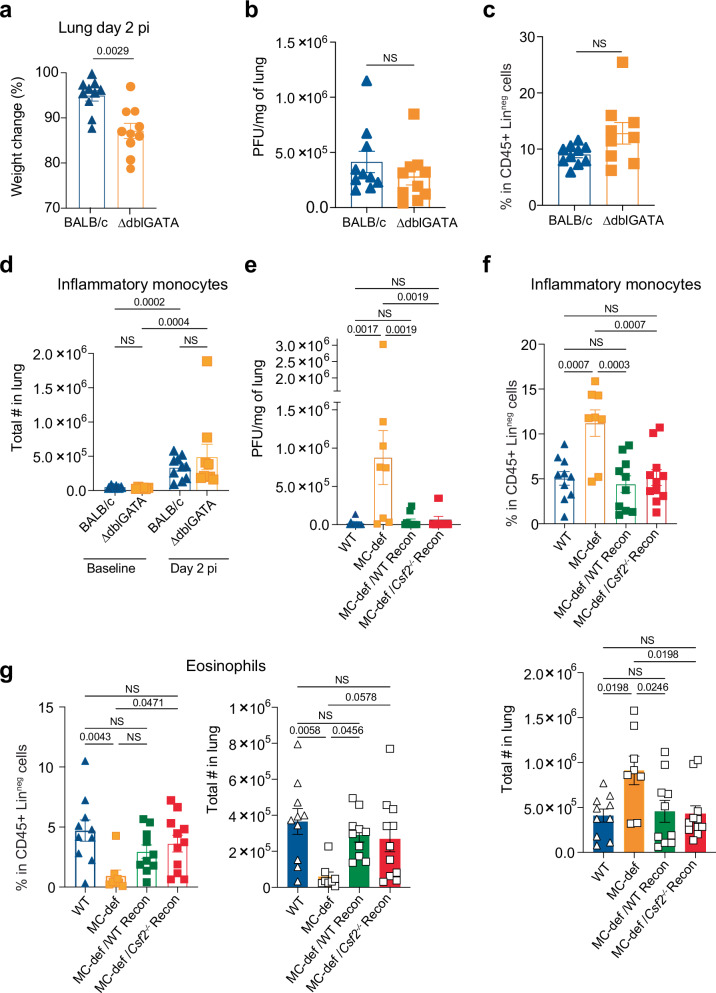
Contributions of eosinophils and mast cell-derived GM-CSF to inflammatory monocyte responses during early RSV infection. Eosinophil-containing BALB/c mice and eosinophil-deficient ΔdblGATA mice were infected intranasally with 4 × 10^6^ PFU RSV and examined on day 2 postinfection (pi). **a**, **b**. Weight loss relative to initial body weight (**a**) and RSV viral loads in lung tissue (**b**) of BALB/c (*n* = 10) versus ΔdblGATA (*n* = 10) mice infected with RSV on day 2 pi. Data were compiled from two experiments and were analyzed by a two-sided Mann–Whitney *U* test. **c**, **d**. Percentage (**c**) and total numbers of inflammatory monocytes (**d**) in lung tissues of RSV-infected BALB/c (*n* = 10) versus ΔdblGATA mice (*n* = 9), compared with uninfected controls (**d**) (*n* = 6, for each strain for total numbers). Data were compiled from two experiments and were analyzed by a two-sided Mann–Whitney *U* test. **e–g**. Mast cell-containing WT mice, mast cell-deficient *Cpa3-Cre; Mcl-1*^*fl/fl*^ (MC-def) mice, and MC-def mice reconstituted with either WT bone marrow-derived mast cells (BMMCs) or *Csf2*^−/−^ BMMCs were infected intranasally with 4 × 10^6^ PFU RSV and analyzed at day 2 pi. Quantification of RSV load in the lung tissue (**e**), percentage and total cell number of inflammatory monocytes (**f**) and eosinophils (**g**) within CD45^+^Lin^neg^ in the lung tissues. WT (*n* = 10), MC-def (*n* = 8), MC-def + WT BMMC (MC-def WT Recon, *n* = 10), and MC-def + *Csf2*^−/−^ BMMC (MC-def *Csf2*^−/−^Recon, *n* = 11). Data were compiled from two experiments and were analyzed using one-way ANOVA with Holm–Sidak’s multiple comparisons test. Data are presented as mean ± SEM in all the graphs. Exact *P* values are displayed in each graph.

To ascertain whether reduced eosinophil frequency, which was associated with mast cell deficiency, contributed to heightened recruitment of inflammatory monocytes during RSV infection, we utilized ΔdblGATA mice, which lack eosinophils^43^. Following intranasal RSV infection, body weight, viral load, and innate immune cell infiltration were assessed on day 2 pi. Analysis revealed significantly reduced weight loss (Fig. 7a) of RSV-infected eosinophil-deficient ΔdblGATA mice compared to BALB/cJ mice. However, we observed no significant differences in viral load between BALB/cJ and ΔdblGATA mice (Fig. 7b), which supports the findings of Phipps et al.^44^, where viral load differences were reduced only after day 3 pi in IL-5Tg mice (which contain more eosinophils) compared to WT mice.

Notably, RSV-infected ΔdblGATA mice did not exhibit increased inflammatory monocytes at day 2 pi when compared with RSV-infected WT controls (Fig. 7c). No differences were found in the percentages and total numbers of Ly6C^high^ monocytes in the lungs between these strains (Fig. 7c, d). Inflammatory monocytes showed significant increases in their percentages and total numbers following RSV infection in both BALB/cJ WT and ΔdblGATA mice compared to uninfected mice (Fig. 7d).

### Mast cell-derived GM-CSF is not a major regulator of eosinophils or monocytes during early RSV infection

After observing that mast cell-derived GM-CSF contributes to maintaining higher eosinophil numbers under steady-state conditions (Fig. 6), we examined whether GM-CSF influenced inflammatory monocyte and eosinophil responses during early RSV infection. To test this, mast cell-deficient *Cpa3-Cre; Mcl-1*^*fl/fl*^ mice were reconstituted with either *Csf2*^−/−^ BMMCs or WT BMMCs and subsequently infected intranasally with RSV. Following RSV infection, WT-BMMC reconstitution was associated with more effective viral control in *Cpa3-Cre; Mcl-1*^*fl/fl*^ mice (Fig. 7e) and normalized both inflammatory monocyte and eosinophil responses (Fig. 7f, g). Reconstitution with *Csf2*^−/−^ BMMCs similarly reduced inflammatory monocyte levels to those observed in WT and WT BMMC-reconstituted mice (Fig. 7f), indicating that mast cell-derived GM-CSF does not contribute substantially to monocyte recruitment during early infection. Importantly, lung eosinophil responses to infection were also restored in both WT BMMC- and *Csf2*^−/−^ BMMC-reconstituted RSV-infected mice to levels comparable with WT controls (Fig. 7g). Together, these findings demonstrate that mast cell-mediated regulation of eosinophils and inflammatory monocytes during early RSV infection can occur largely independently of GM-CSF production.

### Mast cell impact on the nature of T helper cell responses

**Fig. 8:**
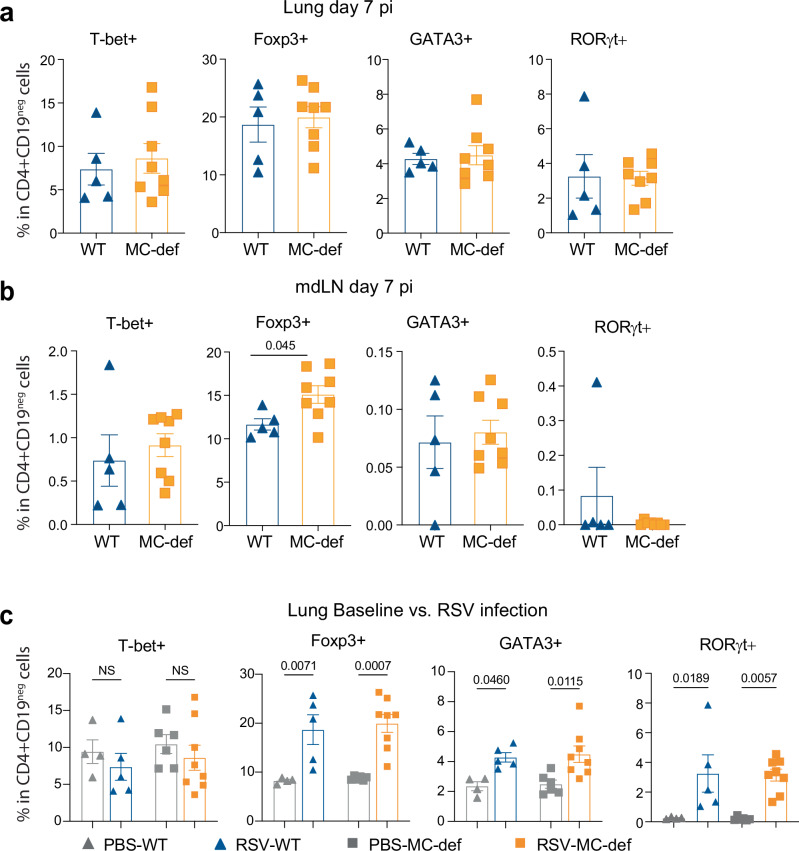
Mast cells do not impact T cell migration and polarization in the lung at day 7 pi. Mast cell-containing littermates (WT) and mast cell-deficient *Cpa3-Cre; Mcl-1*^*fl/fl*^ (MC-def) mice were infected with 4 × 10^6^ PFU RSV intranasally and examined at day 7 postinfection (pi). **a**, **b**. Percentage of T-bet^+^, Foxp3^+^, GATA3^+^, and RORγt^+^ cells within CD4^+^CD19^neg^ populations in lung tissues (**a**) and mediastinal lymph nodes (mdLN) (**b**) at day 7 pi with RSV in WT (*n* = 5) versus MC-def (*n* = 8) mice. Data were compiled from two experiments and were analyzed using a two-sided Mann–Whitney *U* test. **c**. Percentage of T-bet^+^, Foxp3^+^, GATA3^+^, and RORγt^+^ cells within CD4^+^CD19^neg^ populations in lung tissues at baseline, uninfected controls in WT (*n* = 4) versus MC-def (*n* = 6) mice compared with RSV-infected mice displayed in Fig. 8a. Data were compiled from two experiments and were analyzed by one-way ANOVA with Holm–Sidak’s multiple comparisons test. All data are presented as mean ± SEM. Exact *P* values are displayed in each graph. NS indicates nonsignificant values (*P* > 0.05).

To define the role of mast cells in T helper cell polarization during RSV infection, we analyzed major CD4^+^ T cell subsets (Th1, Th2, Th17, and Tregs) in the lungs of RSV-infected WT and mast cell-deficient *Cpa3-Cre; Mcl-1*^*fl/fl*^ mice at day 7 pi using the gating strategy as shown (Supplementary Fig. 1c). No significant differences were observed in the percentage of these subsets between the two groups (Fig. 8a). However, in the mediastinal lymph nodes (mdLN), RSV-infected *Cpa3-Cre; Mcl-1*^*fl/fl*^ mice had a significantly higher percentage of Foxp3^+^ Tregs compared to RSV-infected WT mice (Fig. 8b). RSV-infected mice exhibited an increased proportion of Th cell subsets in lung tissue when compared to their uninfected counterparts (Fig. 8c). These findings suggest that, within the context of RSV infection, mast cells do not exert a substantial impact on the modulation of total T helper effector responses but rather have selective impacts that include critical changes in eosinophil, inflammatory monocyte, and mediator responses that aid in combating RSV infection at early time points pi.

## Discussion

**Fig. 9:**
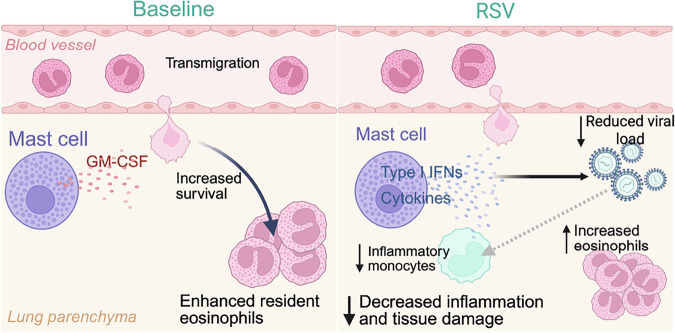
Mast cell function at homeostasis and during early RSV infection. At baseline (left panel), mast cells produce GM-CSF, enhancing the survival and maintenance of resident eosinophils in the lung parenchyma. During early respiratory syncytial virus (RSV) infection (right panel), mast cells release type I interferons (IFNs) and cytokines, which suppress the recruitment of inflammatory monocytes, increase in eosinophil frequencies, and contribute to a reduction in viral load. These coordinated actions reduce inflammation and limit tissue damage in the lung. Illustration created with BioRender.com.

Mast cells play a critical sentinel role at mucosal surfaces, releasing mediators that regulate inflammation and tissue remodeling^6,45^. In this study, the role of mast cells in the early response to RSV infection was examined. The presence of mast cells was associated with decreased early weight loss, decreased RSV viral loads, and reduced airway damage in the initial stages of RSV infection, when compared with responses to infection in the absence of mast cells (Fig. 9). Reconstitution of mast cell-deficient mice confirmed the protective impacts of mast cells, underscoring their importance in controlling early RSV infection and its associated impact on tissue pathology. Additionally, we observed that mast cells promoted eosinophil responses to RSV and modulated eosinophil density and characteristics in uninfected lung tissue, potentially due to mast cell-related changes in GM-CSF levels and IL-25. Overall, our results demonstrate that mast cells are crucial for early RSV host defense, possibly in concert with eosinophils.

Our findings using eosinophil-deficient ΔdblGATA mice demonstrated no significant differences in RSV load or inflammatory monocyte accumulation at day 2 pi, compared to WT controls. These observations remain consistent with a previous study^44^, which showed that eosinophils can contribute to RSV clearance after day 3 pi in both ΔdblGATA and IL-5-Tg mice. Together, these findings demonstrate a critical role for mast cells in the early containment of RSV infection, associated with a key role for mast cells in eosinophil recruitment to the site of RSV infection with a potential later role for eosinophils in the antiviral response.

In the current study, higher levels of local inflammatory cytokines and chemokines were observed in mast cell-deficient mice than in mast cell-containing mice 2 days after RSV infection. Elevated IL-6, TNF, CCL2, CCL4, and CXCL10 levels in mast cell-deficient mice indicate that mast cells are not the major source of this cytokine response and likely reflect the higher viral loads that are observed in mast cell-deficient animals contributing to greater tissue damage^46,47^. Elevated numbers of Ly6C^+^ monocytes were also detected in mast cell-deficient mice, compared with their mast cell-containing littermates in concert with the higher viral load at day 2 pi. While such inflammatory monocytes are critical for antiviral defense, they are also known to exacerbate tissue damage during respiratory viral infections^48,49^. Elevated levels of infection are likely the drivers of increased inflammatory tissue damage. However, we cannot exclude the possibility that the presence of mast cells may more directly reduce inflammatory responses through the release of mediators such as IL-10^50^ or cytokine-degrading proteases^51^.

The precise role of eosinophils in host defence against RSV is unclear. RSV infection induces systemic activation of eosinophils, suggesting their functional involvement in combating RSV-induced respiratory tract disease^44,52^. In contrast, heightened eosinophilic recruitment into the lung in response to formalin-inactivated RSV vaccine antigen^53^ and degranulation of eosinophils in the respiratory tract^54^ supports a role for these cells in the pathogenesis of bronchiolitis. Eosinophils are potent effector cells, producing IFNs and chemokines in response to RSV^44,55^, which can contribute to effective antiviral host defence. Taken together, current data suggest that eosinophils, in concert with mast cells, may have positive early roles in innate defence against RSV infection, while an unrestrained long-term eosinophilic response may also have damaging consequences. Together, these findings demonstrate a critical role for mast cells in the early containment of RSV infection, associated with a key role for mast cells in eosinophil recruitment to the site of RSV infection. Elevated inflammatory monocyte responses during the initial phase of infection likely reflect the greater viral load and subsequent inflammatory response in the absence of mast cells.

Our data also point to potential epithelial and cytokine pathways through which mast cells may promote early antiviral defense. IL-25, a cytokine critical for eosinophil recruitment^32–34^ was elevated in RSV-infected WT but not mast cell-deficient *Cpa3-Cre; Mcl-1*^*fl/fl*^ mice at day 2 pi, correlating with enhanced eosinophil responses in mast cell-containing WT mice. In contrast, the expression of *Il33* and *Tslp*, other epithelial-derived cytokines implicated in type 2 inflammation, remained unchanged between WT and *Cpa3-Cre; Mcl-1*^*fl/fl*^ mice. Additionally, expression of *Muc5b*, a mucin linked to airway protection and viral clearance^35,36^, was increased in mast cell-sufficient mice, in line with reduced RSV burden. These observations suggest that mast cells may enhance mucosal barrier function not only through regulating immune cell recruitment but also by, directly or indirectly, modulating epithelial responses and mucin production during early infection.

Recent studies have identified heterogeneity in intestinal eosinophils, distinguishing basal and active populations based on surface markers^39^. Our findings align with these, demonstrating expression of specific activation markers on subsets of airways eosinophils including PD-L1, CD31, and CD80 associated with inflammation^39,56^. Mast cell-deficient animals exhibited reduced eosinophil density in lung tissue, and the remaining eosinophils had altered PD-L1 expression. These differences were reversed by reconstitution of *Cpa3-Cre; Mcl-1*^*fl/fl*^ animals with mast cells from WT mice, indicating that they were not the result of other potential defects in the mast cell-deficient *Cpa3-Cre; Mcl-1*^*fl/fl*^ strain. Perivascular mast cells are abundant in the airways,can produce eosinophil-recruiting chemokines (CCL11, CXCL10, and CCL5), and are a potent source of cytokines such as GM-CSF that facilitate eosinophil survival and retention in the tissue^37,38^. In uninfected animals, both eosinophils and GM-CSF were observed at higher levels in mast cell-containing lung tissue than in tissues from mast cell-deficient mice. The mast cell mediator 5-hydroxyindoleacetic acid (5-HIAA), which acts as an eosinophil chemoattractant through the GPR35 ligand–receptor system^57^, also has a potential role in this process but has not been examined in viral infections.

Although mast cell-derived GM-CSF contributed to maintaining higher eosinophil numbers under baseline conditions, our in vivo reconstitution studies revealed that GM-CSF was not required for mast cell-mediated regulation of eosinophils or inflammatory monocytes during early RSV infection. When *Cpa3-Cre; Mcl-1*^*fl/fl*^ mice were reconstituted with either WT BMMCs or *Csf2*^−/−^ BMMCs and subsequently infected with RSV, both groups similarly restored control of viral load along with normalization of inflammatory monocyte recruitment. Notably, lung eosinophil responses to RSV infection were also restored in *Csf2*^−/−^ BMMC-reconstituted animals to levels comparable with WT controls, indicating that mast cell-derived GM-CSF is not essential for the mast cell-dependent eosinophil response observed during infection. Mast cells are known to regulate eosinophil recruitment, function, and survival through multiple mediators, including CCL5, CCL11, IL-5, and IL-3. Even when a specific mediator such as GM-CSF is absent, mast cells can still release multiple antivirals and proinflammatory signals that indirectly promote eosinophil infiltration into the lung.

Previous studies have demonstrated the role of mast cells in shaping Th and CD8^+^ T cell responses^58,59^ and in suppressing Treg functions^60,61^. Current literature suggests these effects may involve diverse mechanisms, including the recruitment and maturation of DCs within tissues or draining lymph nodes in response to mast cell-derived mediators, and direct antigen presentation by mast cells to modulate T cell responses^62,63^. Interestingly, in our work, the absence of mast cells did not significantly affect CD4^+^ Th1 or Th2 cell frequencies during RSV infection at day 7 pi, in keeping with observations that their primary influence occurs at early time points when mobilization of local immune effector mechanisms can be critical to contain infection.

Although mast cells produced type I IFNs in response to RSV in vitro, we did not observe enhanced bulk lung type I IFN responses in mast cell-sufficient mice in vivo. Instead, *Ifnb1* expression correlated with viral load and was modestly elevated in mast cell-deficient mice, consistent with an increased response to the presence of increased virus rather than impaired antiviral IFN responses. These findings suggest that mast cells restrict RSV infection through localized, early antiviral mechanisms, which may also occur in the upper airways, rather than by amplifying global, longer term, type I IFN responses in the lung. Consistent with this interpretation, supernatants from RSV-infected human mast cells reduced RSV infection of BEAS-2B airway epithelial cells in vitro, supporting a localized mast cell-mediated antiviral effect at the epithelial interface. Therefore, the mechanisms whereby mast cells limit RSV burden and subsequent infection-associated inflammation are likely complex and multifaceted, going beyond the impacts of RSV-induced mast cell IFN production^64,65^. We have previously demonstrated that the products of virus-infected human mast cells such as CXCL8, CCL3 and CCL5 can recruit NK cells and CD56^+^ T cells^14,15^. Mast cell-derived type I IFNs appear to have a key role in activating NK cells to promote their killing function, in addition to their classical antiviral roles^66^. Moreover, mast cells have been implicated in activating the endothelium through IL-1β and TNF production, promoting the local recruitment of antiviral effector cells^16^, a process documented during primary dengue virus infection^67,68^.

In this study, mast cell roles in RSV infection were primarily examined using young adult (4–6-week-old) mice, an age at which mast cell populations are fully established and lung architecture is stable, enabling clear assessment of mast cell-intrinsic protective functions. Mast cell reconstitution experiments were additionally conducted in older animals, with reconstituted and control mice being approximately 12–14 weeks old at the time of RSV infection. Although neonatal and aged models more closely reflect populations at highest clinical risk for severe RSV disease, developmental and age-associated immune differences complicate mechanistic interpretation. Accordingly, young adult models remain the standard for defining cell-specific antiviral pathways, with extension to neonatal, aged, or human systems representing important future directions.

Taken together, our studies reveal a unique paradigm for the function of mast cells during early RSV infection, where mast cells exhibit protective functions, controlling viral load and limiting tissue damage. We have also identified mast cell-dependent promotion of baseline eosinophil populations, eosinophil recruitment upon infection, and their PD-L1 expression profiles in the airways. Our findings reveal a previously unrecognized role for mast cells, reducing viral loads and limiting subsequent tissue damage during respiratory viral infection. Our current results suggest that preserving resident mast cell populations and function early in infection, could be important for combating virus-induced airways damage and warrants consideration in the context of evolving clinical management strategies for acute RSV infection.

## Methods

### Mice

C57BL/6J (Strain No. 000664), BALB/cJ (Strain No. 000651), B6. *Csf2*^−/−^ /J (Strain No. 026812), and ΔdblGATA (Strain No. 005653) mice were obtained from the Jackson Laboratories (Bar Harbor, ME, USA). *Cpa3-Cre; Mcl-1*^*fl/fl*^ mice (also denoted as mast cell-deficient, MC-def, internally called “Hello Kitty” or “HK” mice)^26^ were a generous gift from Drs. S. Galli and M. Tsai (Stanford University, Palo Alto, CA). These mice were bred in the Carleton Animal Care Facility at Dalhousie University. Mice were housed under specific pathogen-free conditions on a 12-h light/dark cycle at 22  ±  2°C and 40%–60% humidity with food and water provided ad libitum. Experimental and control mice were bred/maintained under similar housing conditions. Experiments were performed using both male and female mice. *Cpa3-Cre; Mcl-1*^*fl/fl*^ and their littermate controls were males in all the experiments and were co-housed, whereas ΔdblGATA and BALB/c mice included both male and female mice. During RSV infection experiments, infected mice were housed separately from uninfected mice. Animal studies reported here were approved by the University Committee on Lab Animals at Dalhousie University (Protocol Nos. 18-042, 22-028, and 25-023).

### Antibodies

Fluorochrome-labeled antibodies against mouse antigens were obtained from BD Biosciences (Mississauga, Canada), BioLegend (San Diego, CA), or Invitrogen (Carlsbad, CA). The antibodies include CD3e, CD4, CD8a, CD11c, CD11b, CD19, CD24, CD25, CD40, CD31, CD44, CD45, CD45R, CD49b, CD80, CD86, CD90.2, CD117, CD127, CD172a, CD206, CD317, Foxp3, F4/80, GATA3, Ly6C, Ly6G, MHCII, MerTK, NK1.1, PD-L1, RORγt, Sca-1, Siglec-F, ST2, and T-bet. Each antibody clone, catalog number, source, and dilution used for staining are provided in Supplementary Table 1.

### Cell preparation, staining, and flow cytometry

Lung samples were prepared for flow cytometry analysis as described^69^. Lung tissues were minced into 1 mm pieces, then digested with 4 mg/mL collagenase D (Cat. No. 11 088 858 001, Roche Diagnostics, Indianapolis, IN) in the presence of 80 µg/mL DNase (Cat. No. 11 284 932 001, Roche Diagnostics) at 37°C for 45 min with rotation in an incubator. The digested samples were passed through a wire mesh, and the cells were centrifuged at 400 × *g* for 6 min at 10°C. To lyse red blood cells, the cell pellet was resuspended in 1 mL of ammonium chloride (ACK lysis) buffer for 3–4 min at room temperature and then washed in 1× PBS. Lung samples that had poor cell recovery in the digestion were not used for the flow cytometry analysis.

For flow cytometry staining, single-cell suspensions from lung digestion, spleen, and lymph nodes, or in vitro cultures were stained with a fixable viability dye (eFluor 780, or 575V) for 30 min in PBS at 4°C, followed by washing in FACS buffer containing 2% FBS (Cat. No. 18140071, Gibco, Thermo Fisher Scientific, Whitby, Canada) and 0.02% sodium azide in PBS. Cells were stained with antibodies in the presence of mouse Fc block or CD16/CD32 (Cat. No. 553142, BD Biosciences) for 30 min at 4°C. The antibody cocktails were prepared in BV Horizon Brilliant Stain Buffer (Cat. No. 566347, BD Biosciences). Samples were then washed twice in FACS buffer, fixed with 1% paraformaldehyde, and resuspended in FACS buffer. Intracellular staining was performed using the Invitrogen FoxP3/Transcription Factor Staining Buffer Set (Cat. No. 00-5523-00, Thermo Fisher) according to the manufacturer’s instructions. Afterward, the cells were washed twice, fixed in 1% paraformaldehyde, and resuspended in FACS buffer for flow cytometry acquisition. Single-color controls were prepared using UltraComp beads (Cat. No. 01-3333-42, Invitrogen). To ensure consistency across experimental flow cytometry acquisition, Sphero™ Rainbow Calibration Beads (Cat. No. 559123, BD Biosciences) were used. Flow cytometry data were collected on a BD LSRFortessa or BD FACSymphony cytometer and analyzed using FlowJo™ software version 10.8.1 (BD Biosciences).

In the flow cytometry analysis, absolute numbers of immune cell subsets were calculated by combining total viable cell counts with subset frequencies determined by flow cytometry. For spleen, lymph nodes, and peritoneal fluid, total cell counts were obtained directly from complete single-cell suspensions and multiplied by subset percentages. For lung samples, enzymatically digested tissue was counted from a defined processed tissue mass, and values were extrapolated to total lung weight to estimate whole-organ cell numbers. Data generated using this method are presented in Figs. 2c–f, 5c, d, 6a–e, g, h, 7c, d, f, g, 8a–c and Supplementary Figs. 2a–c and 4a–e, g, h.

### BMMC generation

BMMCs were cultured from bone marrow harvested from the femurs and tibiae of female C57BL/6 or B6. *Csf2*^−/−^/J mice. Bone marrow was flushed using RPMI containing 10% FBS and passed through a 40 μm cell strainer, then centrifuged at 300 × *g* for 10 min at 4°C. The cells were cultured in media containing 10% FBS, 1× penicillin–streptomycin, 50 mM 2-mercaptoethanol, 200 nM prostaglandin E_2_ (PGE_2_) (Cat. No. 2296, Batch 12B/308531, Tocris Bioscience, Minneapolis, MN, USA), and 15% WEHI-3B cell culture supernatant as a source of IL-3. Cells were replenished with fresh medium every 3–4 days. After 6 weeks, the purity of BMMCs was assessed by flow cytometry staining for CD117 (anti-mouse CD117). BMMCs of >98% purity were used for in vitro and in vivo reconstitution experiments.

### Respiratory syncytial virus

A recombinant GFP-expressing (rg) RSV-224 (a derivative of the A2 strain) was kindly provided by Drs. Mark E. Peeples and Peter Collins at the Nationwide Children’s Hospital Research Institute Center for Vaccines and Immunity (Columbus, OH). RSV was propagated in HEp-2 cells, as previously described^70^, detailed as follows. HEp-2 cells (kindly provided by Dr. Robert Anderson, Dalhousie University, Halifax, Canada) were cultured in DMEM containing 10% FBS. At 30% confluency, cells were infected with rgRSV-224 at a multiplicity of infection (MOI) of ~1 for 2 h at 37°C. The RSV inoculum was removed, and cells were resuspended in fresh 20 mL of DMEM containing 2% FBS and incubated at 37°C for 2 days. The medium was aspirated and replenished with fresh DMEM with 2% FBS and incubated for 24 h. When infected cells showed >90% GFP positivity under a fluorescence microscope, virus purification was initiated.

Culture supernatant was collected and combined with scraped cells, followed by high-speed vortexing to detach viral particles. Cells were disrupted by repeated pipetting up and down, which facilitated the release of virus. The content was centrifuged at 400 × *g* for 10 min at 4°C. Next, 20 mL of culture supernatant was layered over chilled sucrose and centrifuged at 27,000 × *g* for 5 h at 4°C using an SS-34 rotor ultracentrifuge (Sorvall RC6, Thermo Fisher). After discarding the supernatant, the pellet was resuspended in MEM with 10% trehalose. Purified virus aliquots were snap-frozen and stored at −80°C. Viral titration was conducted in HEp-2 cells by GFP fluorescence assessment after 24 h incubation at 37°C.

### RSV infection of mast cells

Mast cells were infected at an MOI of 5–10 in Opti-MEM containing 3 ng/mL rmIL-3 (Cat. No. 575506, BioLegend) for BMMCs, with each condition (mock vs. RSV infection) performed in technical duplicate. After 2 h, cells were washed and resuspended in RPMI containing 2% FBS and 3 ng/mL IL-3 for BMMCs, and soybean trypsin inhibitor (Cat. No. 650357, MilliporeSigma, Burlington, MA) at 100 μg/mL. The inhibitor was included to reduce the proteolytic degradation of mast cell products. At the 24 h time point, BMMCs were collected for flow cytometry analysis or for qPCR, and supernatants were collected for ELISA. Data from this method are presented in Fig. 4a–c.

### RNA extraction, reverse transcriptase reaction, and quantitative PCR (qPCR)

Mast cells and lung tissue samples were collected in TRIzol reagent (Cat. No. T9424, Sigma-Aldrich, Darmstadt, Germany or Cat. No. 15590026, Invitrogen) and RNAlater (Cat. No. R0901, Sigma-Aldrich), respectively, and stored at −80°C until RNA extraction. RNA extraction was performed using the RNeasy Plus Kit (Cat. No. 74106, Qiagen, Germantown, MD) according to the manufacturer’s instructions. Between 150 and 300 ng of purified RNA was used for cDNA synthesis in each reverse transcription reaction. Genomic DNA was removed using the gDNA wipe-out buffer at 42°C for 2 min. cDNA synthesis was then carried out using the Quantiscript RT Kit (Cat. No. 79254, Qiagen), following the manufacturer’s protocol. Commercially available forward and reverse primers (250–500 nM, Qiagen or Bio-Rad, Mississauga, Canada) were used for *Ccl2, Ccl4, Ccl5, Cxcl10, Ccl11, Il1β, Il6, Il18, Tnf, Vegfa, Ifna1, Ifna2, Ifna14, Ifnl2, Ifnl3*, *Ifnb1, Il10, Csf2, Tpsb2, Il33,* and *Il5*. The primers catalog numbers and sources are provided in Supplementary Table 2. Primers for *Gusb, Hprt1, Il25, Tslp, Muc5ac,* and *Muc5b* were designed in-house, produced by IDT Technologies. The primer sequences used were *Tslp* Forward: GCAAATCGAGGACTGTGAGAGC, Reverse: TGAGGGCTTCTCTTGTTCTCCG; *Il25* Forward: TGGCTGAAGTGGAGCTCTGCAT, Reverse: CCCGATTCAAGTCCCTGTCCAA; *Muc5ac* Forward: CCACTTTCTCCTTCTCCACACC, Reverse: GGTTGTCGATGCAGCCTTGCTT; *Muc5b* Forward: CTGAAGACCTGTCGGAACCCAA, Reverse: GCCACACACTTCATCTGGTCCT; *Gusb* Forward: AAGACTGACACCTCCATGTATC, Reverse: TAGAGGACCACAGATCGATGC; and *Hprt1* Forward: TCGTGATTAGCGATGATGAACC, Reverse: CAGTCCTGTCCATAATCAGTCC. Quantitative PCR was performed using 1× SSoAdvanced™ Universal SYBR® Green Supermix (Cat. No. 1725274, Bio-Rad) on an MX3000P qPCR system, and samples were run in technical duplicates. Data were analyzed with CFX Maestro™ software to determine relative gene expression. Data from this method are presented in Figs. 3c, d, 4c, 6f and Supplementary Figs. 2e, 3b, 4f.

### RSV infection of mice

Approximately 4–6-week-old mast cell-deficient *Cpa3-Cre; Mcl-1*^*fl/fl*^ mice and their WT littermates (mast cell-containing, control mice), ΔdblGATA, and BALB/cJ (matched background strain) mice were intranasally infected with RSV. The mice were weighed, anesthetized (ketamine–xylazine combination), positioned on their backs, and received 30–40 μL of diluted RSV (4 × 10^6^ PFU per mouse). The RSV was administered intranasally by adding it drop by drop into each nostril. Control mice received the same volume of sterile PBS. In the BMMC reconstitution experiments, 4–6-week-old *Cpa3-Cre; Mcl-1*^*fl/fl*^ mice that received BMMCs were allowed 7–8 weeks for effective mast cell reconstitution in the lung and other tissues. Mice were weighed and monitored daily until their experimental endpoint. At different time intervals, mice were deeply anesthetized with ketamine–xylazine and euthanized by exsanguination prior to tissue collection in accordance with institutional animal care and biosafety guidelines. Uninfected mice were euthanized under anesthesia followed by CO_2_ inhalation. The lung samples and mediastinal lymph nodes (mdLNs) were harvested for molecular, immunological, and histological analyses. The lung was removed, weighed, and the left lung was cut into two sections. One section was placed in a histology cassette and fixed in 10% neutral formalin. The other section was cut into approximately 3 mm pieces, snap-frozen, and stored at −80°C for cytokine analyses. The upper part of the right lung was placed in 0.5 mL of RNAlater™ solution (Cat. No. R0901, Sigma-Aldrich) and stored at −20°C for analyses of mRNA expression and RSV viral load. The rest of the lung samples were collected for flow cytometry analysis.

### BMMC reconstitution into *Cpa3-Cre; Mcl-1*^*fl/fl*^ mice

In vitro-generated BMMCs derived from C57BL/6 and *Csf2*^−/−^ C57BL/6 mice were transferred into 4–6-week-old *Cpa3-Cre; Mcl-1*^*fl/fl*^ mice (4 × 10^6^ i.p. and 2 × 10^6^ i.v. per mouse). Mice were maintained for 7–8 weeks for effective reconstitution of mast cells in the lung and other tissues^71,72^. These mice were used for experiments along with age-matched WT or *Cpa3-Cre; Mcl-1*^*fl/fl*^ mice. Data from this method are presented in Figs. 5, 6g, 7e–g.

### Luminex

Lung samples were prepared for Luminex analysis by adding 300 µL of 1× Complete Mini, EDTA-free Protease Inhibitor (Roche Diagnostics, Indianapolis, IN) to the collected lung samples, followed by two 10-s sonication pulses at 50% amplitude using a Fisherbrand™ Model 120 Sonic Dismembrator on ice. Subsequently, the sonicated samples were centrifuged at 15,000 × *g* for 15 min at 4°C, and the resulting supernatants were stored at −80°C. The samples (lung lysates and serum), after centrifugation at 16,000 × *g* for 4 min, were then diluted threefold in Calibrator Diluent RD6-52. The analysis was carried out using a Multi-Analyte Mouse Magnetic Luminex Assay (Cat. No. LXSAHM-14 kit, R&D Systems, Minneapolis, MN) and a Bio-Plex™ 200 System (Bio-Rad), following the manufacturer’s instructions. Samples were run in technical duplicates. The assay yielded protein quantification data for mouse CCL2, CCL4, CCL5, CXCL10, IL-1β, IL-4, IL-5, IL-6, IL-13, IL-33, IL-10, IFN-γ, and TNF, which was expressed as pg per mg of tissue. In this assay, the samples with analyte concentrations below the detectable range are reported as the lower limit of detection (LLOD) based on each analyte’s standard curve. Furthermore, same control tissues lysates (PBS-treated) were included for the day 2 and day 7 pi time points data in Fig. 3a, b and Supplementary Fig. 2f, g.

### ELISA

Cytokines and chemokines were quantified in culture supernatants and lung lysates from naïve mice using ELISA kits according to the manufacturers’ instructions. GM-CSF (Cat. No. DY415), IFNβ1 (Cat. No. DY8234-05), and CCL11 (Cat. No. DY420) ELISA kits were obtained from R&D Systems (Minneapolis, MN, USA); CXCL10 from Invitrogen (Cat. No. BMS6018MST); CCL5 from PeproTech (Cat. No. KE10017); and IL-5 from Abcam (Cat. No. Ab204523). Concentrations were calculated from standard curves generated for each analyte following the manufacturers’ recommended protocols. Data from this method are presented in Figs. 4c and6f.

### Droplet digital PCR

The cDNA was added to a 20 µL ddPCR reaction containing EvaGreen Supermix (Cat. No. 1864033, Bio-Rad) and 125 µM primers designed for the viral N protein (forward: 5′-AAGATCAACTTCTGTCATCCAGC-3′ and reverse: 5′-CTGCACATCATAATTAGGAGTATC-3′). The mixture was randomly partitioned into 20,000 droplets using the QX200 Droplet Generator (Bio-Rad), then amplified for 50 cycles under optimized PCR conditions. Positive and negative fluorescent droplets were counted using the QX200 Droplet Reader (Bio-Rad), and the concentration of target amplicons was determined using QuantaSoft software (Bio-Rad). A serial dilution of cDNA from an RSV stock of known titer was amplified by ddPCR as described above, and the standard curve was used to calculate the absolute count of RSV genome copies per mg of lung tissue. Data from this method are presented in Figs. 1b, c, 5b, and 7b, e.

### Histology scoring

Histological evaluation was conducted using the ASL/ALI scoring system^27,28^. Approximately four to five random fields of lung tissue at 200× total magnification were selected and assessed in a blinded fashion by two independent evaluators according to the ALI scoring system^27,28^. The following parameters were examined: (a) immune cell infiltration in the alveolar space (none = 0, 1–5 cells = 1, >5 cells = 2); (b) immune cell infiltration in the interstitial space/septae (none = 0, 1–5 cells = 1, >5 cells = 2); (c) presence of hyaline membranes (none = 0, one membrane = 1, >1 membrane = 2); (d) presence of proteinaceous debris in air spaces (none = 0, one instance = 1, >1 instance = 2); (e) alveolar septal thickening relative to mock controls (<2× mock thickness = 0, 2–4× thickness = 1, >4× thickness = 2). Scores were calculated using the formula [(20 × a) + (14 × b) + (7 × c) + (7 × d) + (2 × e)]/100. Final scores were determined by averaging the scores from four to five fields per mouse. Histology images were taken using a Zeiss Axio Observer microscope and Zen Blue 3.0 software. Data from this method are presented in Fig. 1e.

### Culture of human CBMCs and RSV infection

Umbilical cord blood samples were collected with informed consent under approval from the Izaak Walton Killam Health Centre Research Ethics Board (Protocol No. 1005110). CBMCs were generated from mononuclear cells^73,74^. Mature CBMCs (>95% purity) were seeded at 1 × 10^6^ cells/mL in mast cell growth medium containing 100 ng/mL stem cell factor (SCF, Cat. No. BT-SCF-01M, R&D Systems) and 10 ng/mL IL-6 (Cat. No. 570808, BioLegend) for 24 h, after which the cells were transferred to activation medium containing 10 ng/mL SCF and no IL-6 for 18 h prior to infection. CBMCs were infected with RSV at an MOI of 5 for 2 h at 37°C in serum-free RPMI 1640 supplemented with 20 mM HEPES and 10 ng/mL SCF. Cells were then washed in serum-free RPMI and resuspended in activation medium (RPMI 1640 supplemented with 20 mM HEPES, 10 ng/mL SCF, 1% FCS, and 100 μg/mL soybean trypsin inhibitor) and incubated for 24 h. Supernatants were collected, clarified, and stored at −80°C for downstream analyses.

### BEAS-2B cell culture

BEAS-2B human bronchial epithelial cells (Cat. No. CRL-3588, ATCC) were maintained in Bronchial Epithelial Cell Basal Medium (BEBM, ATCC, Cat. No. CC3170, Lonza) supplemented with the complete SingleQuot kit (Cat. No. CC417570, Lonza), excluding gentamicin–amphotericin, according to the manufacturer’s recommendations. Tissue culture flasks were precoated with a matrix solution containing 0.01 mg/mL fibronectin (in-house purified), 0.03 mg/mL bovine collagen type I (Cat. No. C4243-20 mL, Sigma-Aldrich), and 0.01 mg/mL BSA (Cat. No. 107 350 94001, Roche Diagnostics) diluted in BEBM. Coated T-75 flasks were incubated overnight at 37°C, aspirated before use, and used immediately or stored at 4°C for up to 1 month. Cells were thawed, washed in BEBM (200 × *g*, 10 min), and seeded at 0.1–0.3 × 10^6^ cells per T-75 flask. For passaging, sub-confluent monolayers were gently detached using 0.25% trypsin–0.53 mM EDTA (Cat. No. 12604-021 or 25200-072, Gibco), neutralized with BEBM, pelleted (300 × *g*, 10 min), and reseeded at the desired density. Data from this method are presented in Fig. 4d.

### Effect of RSV-stimulated CBMC supernatants on BEAS-2B infection

To assess whether mast cell-derived soluble mediators modulate RSV infection of airway epithelial cells, BEAS-2B cultures were seeded at 2 × 10^4^ cells per well in matrix-coated 96-well plates and allowed to reach 80–90% confluence overnight. Cell-free supernatants from mock-treated or RSV-infected CBMCs were added to epithelial monolayers concurrently with RSV (MOI 0.1). Cultures were incubated for 20–22 h at 37°C. Infection efficiency was quantified by fluorescence microscopy using GFP-expressing RSV. Wells containing 10–300 fluorescent foci were included for comparative analyses. All experimental conditions were performed in technical triplicate, and antiviral activity was calculated as the percentage reduction in GFP^+^ cells relative to mock-treated CBMC supernatant controls. Data from this method are presented in Fig. 4d.

### Toluidine blue staining for mast cells

Paraffin-embedded tissue sections (5 µm) were deparaffinized in two changes of xylene (5 min each), followed by two changes of absolute ethanol (5 min each) and one change of 70% ethanol (3 min), and then rinsed in distilled water. Slides were briefly rinsed in 0.33 N HCl and stained in 0.5% toluidine blue (Cat. No. 470302-956, VWR) prepared in 0.33 N HCl for 2 days. Sections were then rinsed twice in 0.33 N HCl, followed by a rinse in tap water. Lung sections, but not tracheal sections, were briefly counterstained with weak eosin Y (two dips), rapidly dehydrated through graded ethanol (70%, 95%, and two changes of 100%), cleared in xylene, and mounted with DPX mounting for histology (Cat. No. 06522, Sigma-Aldrich). Mast cells were manually counted across the entire section, then the total tissue area of each section was measured using ImageJ (version 2.14.0). Mast cell density was expressed as number of cells per mm^2^ of lung tissue. Data from this method are presented in Supplementary Fig. 2c, d.

### Statistical analyses

The sample sizes displayed in the figures correspond to the total number of mice examined, pooled from multiple experiments. Data were analyzed in GraphPad Prism version 6-10 using Mann–Whitney *U* test, Wilcoxon matched-pairs signed-rank test, one-way ANOVA, or two-way ANOVA followed by post hoc analyses, as appropriate for data distribution and experimental design. *P* values < 0.05 were considered statistically significant. Nonsignificant differences are indicated as NS (*P* > 0.05) in the figures. Exact *P* values for significant comparisons are provided in each figure panel.

### Reporting summary

Further information on research design is available in the Nature Portfolio Reporting Summary linked to this article.

## Supplementary information

Supplementary Information

Reporting Summary

Transparent Peer Review file

## Source data

Source Data

## Data Availability

All data supporting the findings of this study are included in the Article and its Supplementary Information or are available from the authors upon reasonable request. The raw numerical data underlying charts and graphs are provided in the Source Data file. Unique reagents generated in this study are available from the authors. Source data are provided with this paper.
