# Supplementary material for: Mast cells support lung eosinophil homeostasis and the acute innate immune response to respiratory syncytial virus
Source: Nat Commun. 2026 May 24;17:6776. doi: 10.1038/s41467-026-73438-w (PMC13385834; doi:10.1038/s41467-026-73438-w)
Supplement: Supplementary file 1 — Supplementary Information [file 41467_2026_73438_MOESM1_ESM.pdf]

Mast cells support lung eosinophil homeostasis and the acute innate immune response to Respiratory Syncytial Virus

Roopa Hebbandi Nanjundappa[1], Christopher R. Liwski[1], Alexander Edgar[1], Matthieu Castonguay[2], Ian D. Haidl[1], Jean S. Marshall[1, 2, 3]*

[1]Department of Microbiology and Immunology, Dalhousie University, 5850 College Street, Halifax, NS, Canada.

[2]Department of Pathology, Dalhousie University, 5850 College Street, Halifax, NS, Canada.

[3]Beatrice Hunter Cancer Research Institute, Halifax, NS, Canada.

*Correspondence: Jean S. Marshall (jean.marshall@dal.ca)

Inventory of Supporting Information:
Supplementary Figure 1
Supplementary Figure 2
Supplementary Figure 3
Supplementary Figure 4
Supplementary Table 1
Supplementary Table 2
Supplementary Table 3
Source Data File provided as a separate Excel document

a

Flow cytometry gating strategy for different immune cells

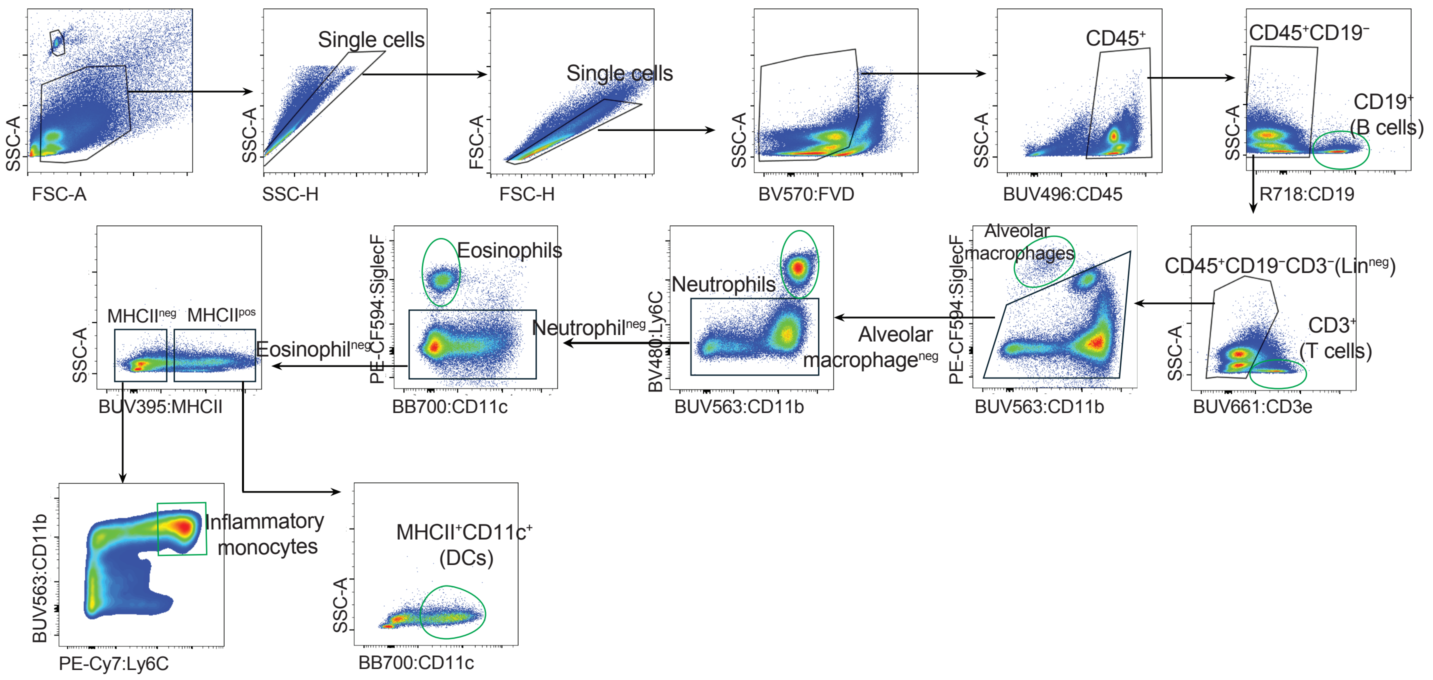

b

Flow cytometry gating strategy for ILC2

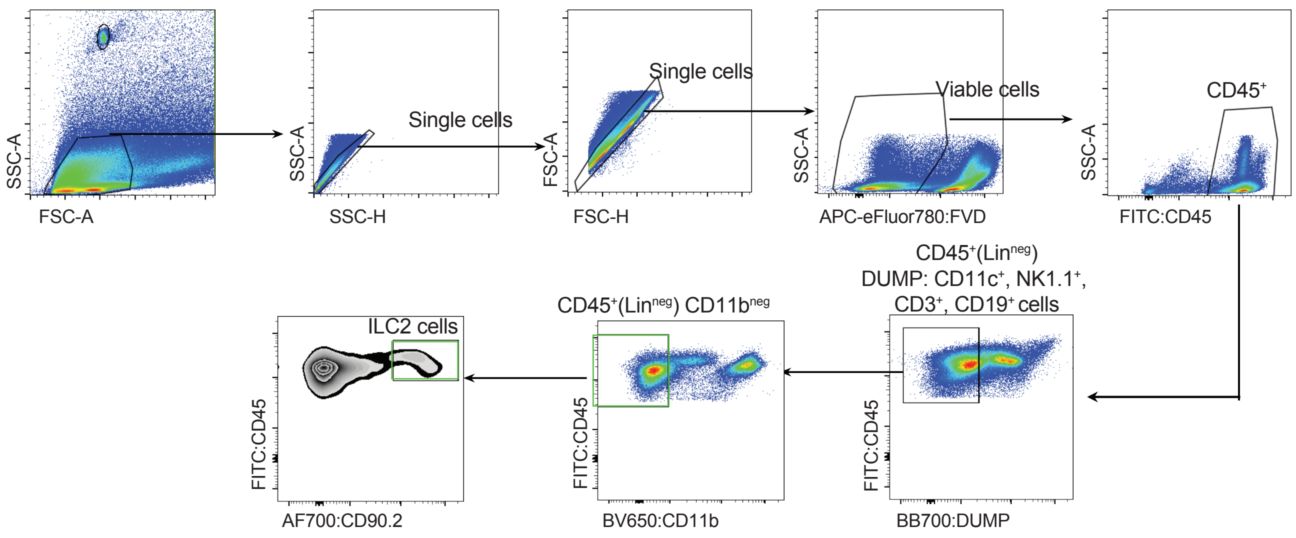

c

Flow cytometry gating strategy for CD4+ T helper subsets

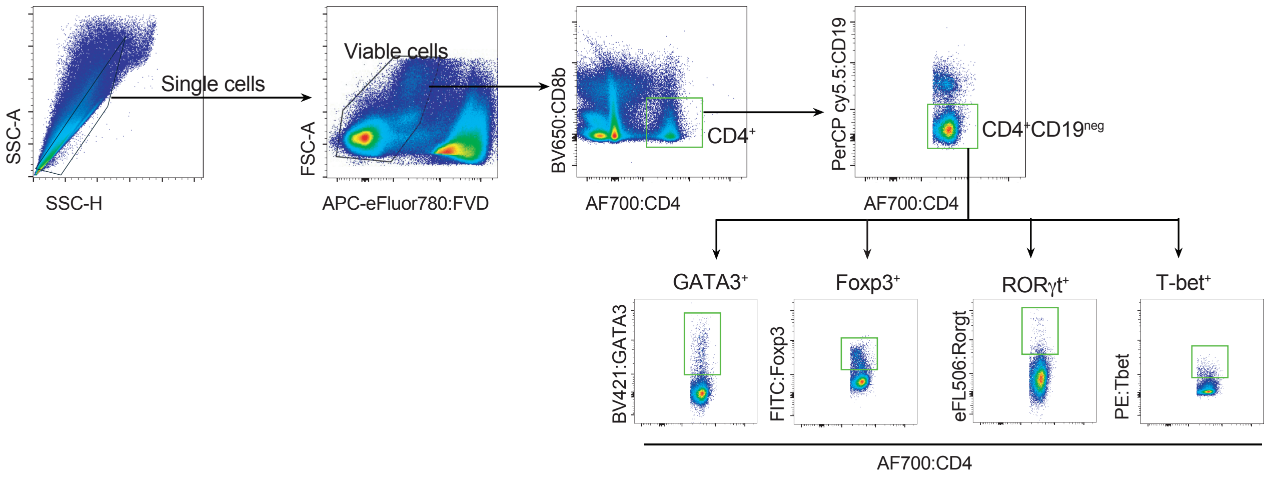

Supplementary Figure 1: Schematics depicting the workflow for flow cytometry

a. Flow cytometry gating strategy for different innate immune cells. Lineage-negative cells were defined as $CD45^{+}CD19^{-}CD3^{-}$. B cells were identified as $CD45^{+}CD19^{+}$; T cells as $CD45^{+}CD3^{+}$; alveolar macrophages as Siglec-F^{high}; neutrophils as $Ly6G^{+}CD11b^{+}$; eosinophils as Siglec-$F^{+}CD11c^{-}$; inflammatory monocytes as $MHCII^{-}$ $CD11b^{+}Ly6C^{high}$; dendritic cells (DCs) as $MHCII^{+}CD11c^{+}$. This strategy was used to analyze the data presented in **Fig. 2c–f, 5c–d, 6a–b, 6d–g, 7c–d, f–g, and Supplementary Figs. 2a–c and 4a**.

b. Flow cytometry gating strategy for innate lymphoid cell type 2 (ILC2). Lineage-negative cells were defined as $CD45^{+}CD11c^{-}CD11b^{-}NK1.1^{-}CD3^{-}CD19^{-}$ and ILC2 were identified as $CD45^{+}CD90.2^{+}$ cells, then confirmed for the expression of ST2. This strategy was used to analyze the data presented in Supplementary **Fig. 4g–h**.

c. Flow cytometry gating strategy for T helper subsets. Cells were first gated on the lymphocyte population, followed by exclusion of dead cells, then $CD4^{+}$ T cells were selected while excluding $CD8^{+}$ cells, and $CD19^{-}CD4^{+}$ cells were then gated to exclude B cells. Within the $CD4^{+}CD19^{-}$ population, T helper cell subsets were identified based on intracellular transcription factor expression **(**$GATA3^{+}$**,** $Foxp3^{+}$**,** $ROR\gamma t^{+}$, and T-bet^{+}). This strategy was used to analyze the data presented in **Fig. 8a–c**.

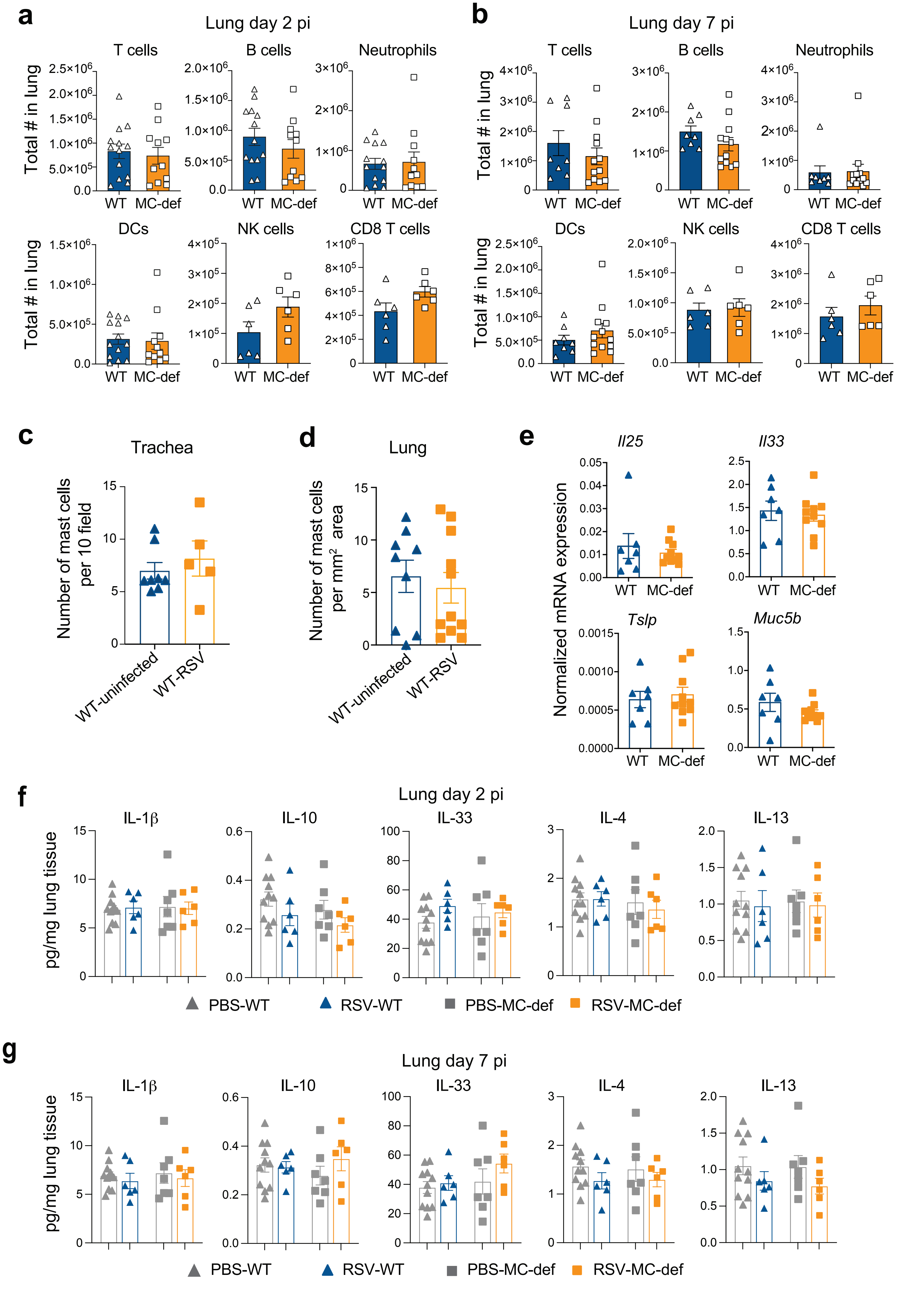
a
Lung day 2 pi
T cells
B cells
Neutrophils
DCs
NK cells
CD8 T cells
Total # in lung
WT MC-def
b
Lung day 7 pi
c
Trachea
Number of mast cells per 10 field
WT-uninfected
WT-RSV
d
Lung
Number of mast cells per mm2 area
e
Il25
Il33
Tslp
Muc5b
Normalized mRNA expression
f
Lung day 2 pi
IL-1β
IL-10
IL-33
IL-4
IL-13
pg/mg lung tissue
PBS-WT
RSV-WT
PBS-MC-def
RSV-MC-def
g
Lung day 7 pi

Supplementary Figure 2: Mast cells modulate early innate immune responses to RSV
Mast cell-containing wildtype (WT) and mast cell-deficient *Cpa3-Cre; Mcl-1*$^{fl/fl}$ (MC-def) mice were infected with 4 $\times 10^6$ PFU RSV intranasally and examined at day 2 and day 7 post-infection (pi).

a. Absolute numbers of T cells, B cells, neutrophils, DCs, NK cells, and CD8$^+$ T cells in lung tissue of WT and mast cell-deficient mice infected with RSV at day 2 pi for mice shown in Fig. 2c-d. T cells, B cells, neutrophils, and DCs: WT (n=13), MC-def (n=11); NK cells and CD8$^+$ T cells: WT (n=6), MC-def (n=6). Each symbol represents one biologically independent mouse. Data were compiled from three experiments and were analyzed by a two-sided Mann–Whitney U test.

b. Absolute numbers of T cells, B cells, neutrophils, DCs, NK cells, and CD8$^+$ T cells in lung tissue of WT and mast cell-deficient mice infected with RSV at day 7 pi for mice shown in Fig. 2e-f. T cells, B cells, neutrophils, and DCs: WT (n=8), MC-def (n=12); NK cells and CD8$^+$ T cells: WT (n=6), MC-def (n=6). Data were compiled from two experiments and were analyzed by a two-sided Mann–Whitney U test.

c. Number of mast cells in the trachea assessed by toluidine blue staining under a wide-field microscope (×125 magnification). WT uninfected (n=8) and WT RSV-infection (n=5). Data were compiled from two experiments and were analyzed by a two-sided Mann–Whitney U test.

d. Number of mast cells in the lung parenchyma assessed by toluidine blue staining. WT uninfected (n=9) and RSV infection (n=11). Data were compiled from two experiments and were analyzed by a two-sided Mann–Whitney U test.

e. Baseline lung gene expression analysis for *Il25, Il33, Tslp, and Muc5b.* WT (n = 7), MC-def (n = 10). Data were compiled from two experiments and were analyzed by a two-sided Mann–Whitney U test.

f–g. Profiling of cytokines and chemokines in the lung tissues from WT and mast cell-deficient mice that were either RSV-infected or PBS-treated, analyzed on day 2 pi (f) and day 7 pi (g), corresponding to the mice shown in **Fig. 3a-b**. PBS-WT (n=11), RSV-WT (n=6), PBS-MC-def (n=7), RSV-MC-def (n=6) at each post-infection time point. Data were compiled from two experiments and were analyzed by one-way ANOVA with Holm-Sidak multiple comparisons test.

Data are presented as mean ± SEM in all the graphs. Exact P values are indicated in each graph where significant. Source data are provided as a Source data file.

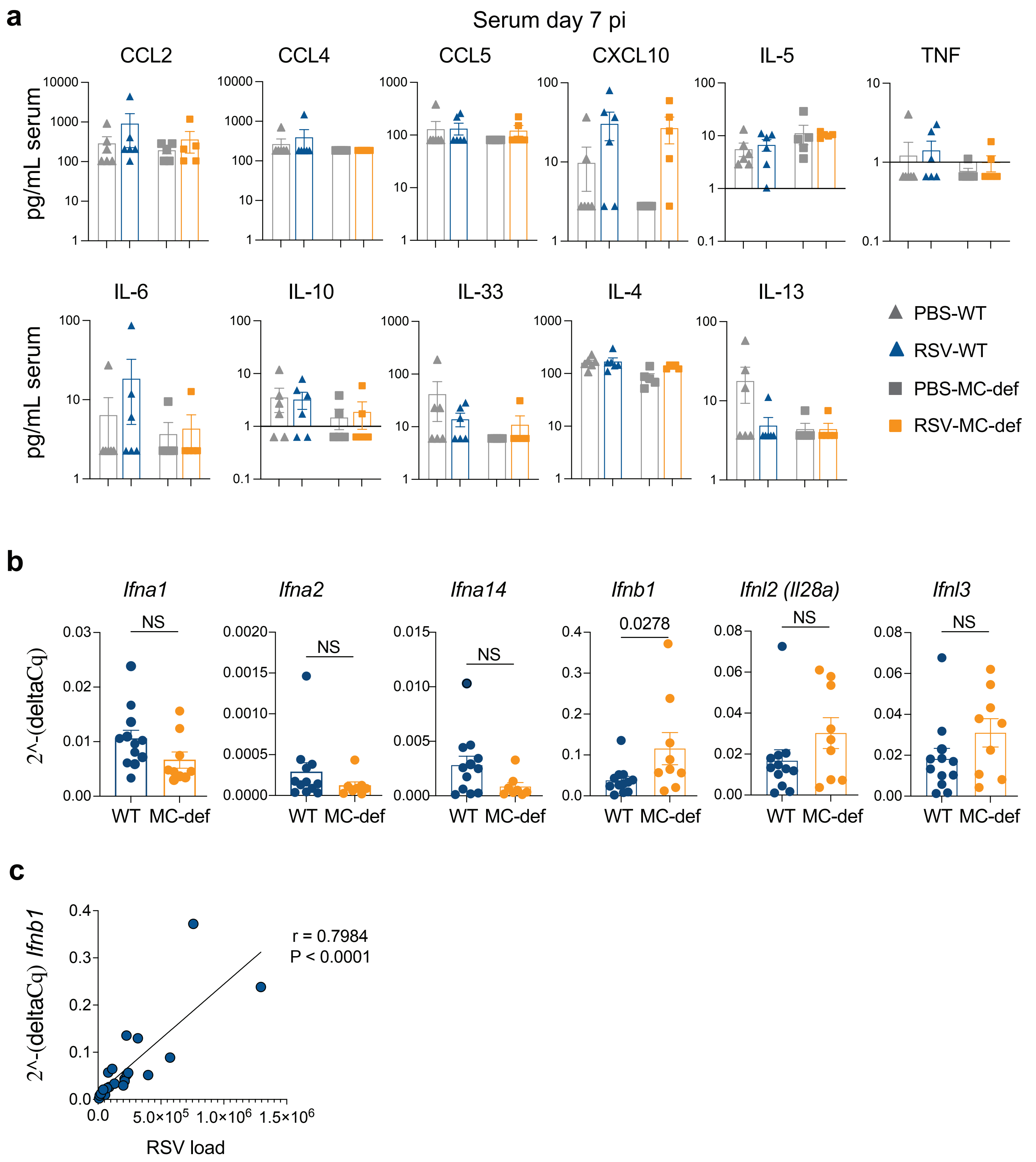
a
Serum day 7 pi
CCL2
CCL4
CCL5
CXCL10
IL-5
TNF
pg/mL serum
IL-6
IL-10
IL-33
IL-4
IL-13
pg/mL serum
PBS-WT
RSV-WT
PBS-MC-def
RSV-MC-def
b
Ifna1
Ifna2
Ifna14
Ifnb1
Ifnl2 (Il28a)
Ifnl3
2^-(deltaCq)
NS
NS
NS
0.0278
NS
NS
WT MC-def
c
2^-(deltaCq) Ifnb1
r = 0.7984
P < 0.0001
RSV load

Supplementary Figure 3: Systemic cytokine responses and lung interferon expression following RSV infection.

a. Profiling of cytokines and chemokines in serum of mast cell-containing wildtype (WT) versus mast cell-deficient *Cpa3-Cre; Mcl-1*$^{fl/fl}$ (MC-def) mice infected with RSV or treated with PBS on day 7 post-infection (pi). PBS-WT (n=6), RSV-WT (n=6), PBS-MC-def (n=5), RSV-MC-def (n=5). Data were compiled from two experiments and were analyzed by one-way ANOVA with Holm-Sidak multiple comparisons test.

b. Normalized lung expression of type I and type III interferon (IFN) genes in WT and mast cell–deficient mice at day 2 post-RSV infection. (WT, n=12; MC-def, n=9). Data were compiled from two experiments and were analyzed by a two-sided Mann–Whitney U test.

c. Correlation between lung *Ifnb1* transcript levels and RSV viral load at day 2 pi. Each point represents an individual mouse. Data were derived from mice shown in **Fig. S3b**, including RSV-infected WT and mast cell-deficient mice with corresponding viral load and *Ifnb1* transcript levels. Data were compiled from three independent experiments and analyzed using Pearson correlation analysis**.** Data are presented as mean ± SEM in all the graphs. Exact P values are indicated in each graph where significant (NS= not significant). Source data are provided as a Source data file.

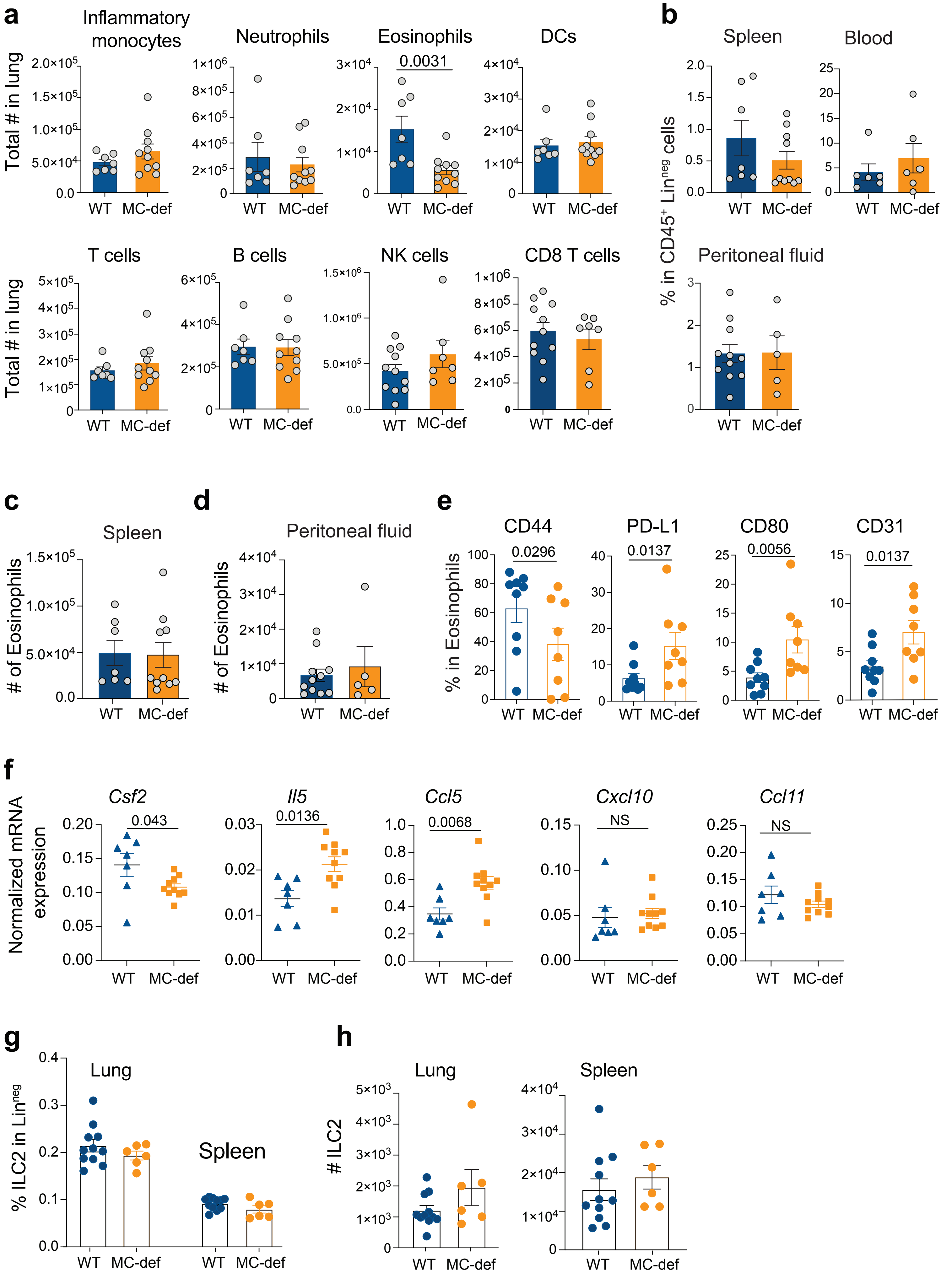

a
Inflammatory monocytes
Neutrophils
Eosinophils
DCs
Total # in lung
0.0031
WT MC-def
T cells
B cells
NK cells
CD8 T cells
b
Spleen
Blood
Peritoneal fluid
% in CD45+ Linneg cells
c
Spleen
# of Eosinophils
d
Peritoneal fluid
e
CD44
PD-L1
CD80
CD31
% in Eosinophils
0.0296
0.0137
0.0056
0.0137
f
Csf2
Il5
Ccl5
Cxcl10
Ccl11
Normalized mRNA expression
0.043
0.0136
0.0068
NS
g
Lung
Spleen
% ILC2 in Linneg
h
ILC2

Supplementary Figure 4: Mast cells influence eosinophil, but not ILC2, counts in the lung. Uninfected mast cell-containing wildtype (WT) and mast cell-deficient *Cpa3-Cre; Mcl-1$^{fl/fl}$* (MC-def) mice were examined at 8-10 weeks of age for immune cells in spleen, peritoneum, lung and blood.

a. Total counts of inflammatory monocytes, neutrophils, eosinophils, DCs, T cells, B cells, NK cells and $CD8^{+}$ T cells in the lung tissue of WT versus mast cell-deficient mice. Inflammatory monocytes, neutrophils, eosinophils, DCs, T cells, and B cells: WT (n=7), MC-def (n=10); NK cells and $CD8^{+}$ T cells: WT (n=11), MC-def (n=7). Each symbol represents one biologically independent mouse. Data were compiled from two experiments and were analyzed by a two-sided Mann–Whitney U test.

b. Percentage of eosinophils in the spleen (WT, n=7; MC-def, n=10), peritoneum (WT, n=11; MC-def, n=5) and blood (WT, n=6; MC-def, n=6) at baseline. Data were compiled from two experiments and were analyzed by a two-sided Mann–Whitney U test.

c–d. Total counts of eosinophils in the spleen, WT, n=7; MC-def, n=10 (c) and peritoneum, WT, n=11; MC-def, n=5 (d) at baseline. Data were compiled from two experiments and were analyzed by a two-sided Mann–Whitney U test.

e. Percentage of CD44, PD-L1, CD80, CD31, and Sca-1 expressing eosinophils in the spleen of WT (n=9) versus MC-def (n=8) mice. Data were compiled from two experiments and were analyzed by a one-sided Mann–Whitney U test.

f. Quantification of normalized gene expression levels of *Csf2, Il5, Ccl5, Cxcl10* and *Ccl11* (lower panel) in the lung tissues of WT (n=7) versus MC-def (n=10) mice. Data were compiled from two experiments and were analyzed by a two-sided Mann–Whitney U test.

g. Percentage of ILC2 populations in the lung (WT, n=11; MC-def, n=6) and spleen (WT, n=11; MC-def, n=6) at baseline. Data were compiled from two experiments and were analyzed by a two-sided Mann–Whitney U test.

h. Total numbers of ILC2 in the lung and spleen for the mice in (Supplementary **Fig.4g**). Data were compiled from two experiments and were analyzed by a two-sided Mann–Whitney U test. Data are presented as mean ± SEM. Exact P values are indicated in each graph where significant (NS= not significant). Source data are provided as a Source data file.

Supplementary Table 1: Antibodies and flow cytometry reagents used in this study

Antibody (Fluorochrome)	Clone	Supplier	Catalogue number	Dilution
CD45R/B220-BV786	RA3-6B2	BD Biosciences	563894	1 in 600
Ly6G-BV480	IA8	BD Biosciences	746448	1 in 200
CD172a-BV421	P84	BD Biosciences	740071	1 in 300
CD11c-BB700	HL3	BD Biosciences	566504	1 in 200
Ly6C-PE-Cy7	HK1.4	BioLegend	128018	1 in 1400
Siglec-F-PE-CF594	E50-2440	BD Biosciences	562757	1 in 200
CD8a-APC-H7	53-6.7	BD Biosciences	560182	1 in 200
CD8a-BV650	53-6.7	BD Biosciences	563234	1 in 200
CD19-R718	ID3	BD Biosciences	567063	1 in 800
CD11b-BUV563	M1/70	BD Biosciences	741242	1 in 1000
I-A/I-E-BUV395	2G9	BD Biosciences	743876	1 in 1500
CD3e-BUV661	145-2C11	BD Biosciences	750638	1 in 200
CD45-BUV496	30-F11	BD Biosciences	569673	1 in 600
CD4-APC	RM4.5	BioLegend	100516	1 in 200
CD11b-BV650	M1/70	BioLegend	101259	1 in 600
CD11c-APC	N418	Invitrogen	17-0114-82	1 in 200
CD3e-BB700 (Dump)	145-2C11	BD Biosciences	566495	1 in 200
CD19- PerCP Cy5.5 (Dump)	6D5	BioLegend	115534	1 in 200
NK1.1-PerCP Cy5.5 (Dump)	PK136	BioLegend	108728	1 in 200
CD24-BV711	M1/69	BD Biosciences	563450	1 in 1200
CD25 PE-CY7	LG.7F9	Invitrogen	25-0271-82	1 in 200
CD40-BUV737	3/23	BD Biosciences	741749	1 in 300
CD45-FITC	30-F11	BioLegend	103108	1 in 400
CD31-BV421	390	BD Biosciences	563356	1 in 200
CD44-APC-eF780	IM7	Invitrogen	47-0441-82	1 in 200
CD45-AF700	30-F11	BD Biosciences	560510	1 in 200
CD49b-PE	DX5	BioLegend/ Invitrogen	108908<br>61-5971-82	1 in 200
CD80-FITC	16-10A1	eBioscience	11-0801-85	1 in 200
CD90.2-AF700	30-H12	BioLegend	105320	1 in 200
CD117-PE	2B8	eBioscience	12-1171-82	1 in 200
CD127-APC	A7R34	eBioscience	17-1271-82	1 in 600
CD206-AF647	MR5D3	BD Biosciences	565250	1 in 200
CD317-BV650	927	BD Biosciences	747605	1 in 100
FoxP3-AF488	150D	BioLegend	320012	1 in 100
F4/80-BUV805	T45-2342	BD Biosciences	749282	1 in 300
GATA-3-BV421	L50-823	BD Biosciences	563349	1 in 100
MerTK-PE	2B10C42	BioLegend	151506	1 in 200
PD-L1-PE	4C7	BioLegend	144203	1 in 100
Rorγt-BV510	Q31-378	BD Biosciences	567177	1 in 100
Sca-1-BV786	D7	BD Biosciences	563991	1 in 200

ST2-PE	DIH4	BioLegend	146607	1 in 200
T-bet-PE	4B10	BD Biosciences	561265	1 in 100
CD16/32 (Fc Block)	2.4G2	BD Biosciences	553142	1 in 200
eBioscience Fixable viability dye eFluor 780		Invitrogen	65-0865-14	1 in 800
BD Horizon Fixable viability stain 575V		BD Biosciences	565694	1 in 5000
BV Horizon Brilliant buffer		BD Biosciences	566347	
Invitrogen UltraComp beads		Invitrogen	01-3333-42	
Sphero^{TM} Rainbow Calibration particles		BD Biosciences	559123	

Supplementary Table 2: Primers used in the study

**Mouse gene name**	**Catalogue No. or details**	**Source**
Gusb (in house)	Sequences provided in the Methods section	Integrated DNA Technologies (IDT)
Hprt1(in house)	Sequences provided in the Methods section	Integrated DNA Technologies (IDT)
Tslp (in house)	Sequences provided in the Methods section	Integrated DNA Technologies (IDT)
Il25 (in house)	Sequences provided in the Methods section	Integrated DNA Technologies (IDT)
Muc5b (in house)	Sequences provided in the Methods section	Integrated DNA Technologies (IDT)
Muc5ac (in house)	Sequences provided in the Methods section	Integrated DNA Technologies (IDT)
Ccl2	qMmuCED0048300	Bio-Rad Laboratories
Ccl4	qMmuCED0044850	Bio-Rad Laboratories
Ccl5	qMmuCED0021047	Bio-Rad Laboratories
Cxcl10	mm.PT.58.43575827	Integrated DNA Technologies (IDT)
Ccl11	PPM02967G	Qiagen
Il1b	PPM031209F	Qiagen
Il6	mm.PT.58.10005566	Integrated DNA Technologies (IDT)
Il18	PPM03112B	Qiagen
Tnf	PPM03113G	Qiagen
Vegfa	qMmuCED0040260	Bio-Rad Laboratories
Ifna1	qMmuCED0061481	Bio-Rad Laboratories
Ifna2	qMmuCED0061426	Bio-Rad Laboratories
Ifna14	qMmuCED0001471	Bio-Rad Laboratories
Ifnl2	qMmuCED0041068	Bio-Rad Laboratories
Ifnl3	PPM34810A	Qiagen
Ifnb1	mm.PT.58.30132453.g	Integrated DNA Technologies (IDT)
Il10	PPM03017C	Qiagen
Csf2	qMmuCED0044875	Bio-Rad Laboratories
Tpsb2	PPM03182E	Qiagen
Il33	PPM32527A	Qiagen
Il5	PPM03014F	Qiagen

Supplementary Table 3: Details of key reagents used in this study.

**Reagents**	**Manufacturer**	**Product number**	**Applications**
	**ELISA kit/Luminex kit**		
GM-CSF	R&D Systems	DY415	
IFNβ1	R&D Systems	DY8234-05	
CCL11	R&D Systems	DY420	
CXCL10	Invitrogen	BMS6018MST	
CCL5	PeproTech	KE10017	
IL-5	Abcam	Ab204523	
Multi-analyte mouse magnetic luminex assay	R&D Systems	LXSAHM-14 kit	
	**Genotyping**		
Cpa3-Cre; Mcl-1$^{fl/fl}$<br>MCL1-R<br>MCL1-F<br><br>CPA3-CRE F<br>CPA3-CRE R	 5'-CTGAGAGTTGTACCGGACAA-3'<br>5'-GCAGTACAGGTTCAAGCCGATG-3'<br><br>5'-CGATGCAACGAGTGATGAGG-3'<br>5'-GCATTGCTGTCACTTGGTCGT-3'		
ΔdblGATA	Flow cytometry confirmation for absence of Siglec-F^{+} cells		
RSV N protein (ddPCR)<br>Forward<br>Reverse	 5'-AAGATCAACTTCTGTCATCCAGC-3'<br>5'CTGCACATCATAATTAGGAGTATC-3		
	**Cell line/ Primary cell**		
Hep-2 cells	Provided by Dr. Robert Anderson, Dalhousie University, Canada (ATCC)	CCL-23	
BEAS-2B cells	ATCC	CRL-3588	
Bone marrow-derived mast cells (BMMC)	Bone marrow isolated from wild-type or *csf2-/-* mice	Primary cells	
Human umbilical cord blood cells	Obtained from human umbilical cord blood samples, to generate cord blood-derived mast cells	Primary cells	
