# Supplementary material for: Mast cells support lung eosinophil homeostasis and the acute innate immune response to respiratory syncytial virus
Source: Nat Commun. 2026 May 24;17:6776. doi: 10.1038/s41467-026-73438-w (PMC13385834; doi:10.1038/s41467-026-73438-w)
Supplement: Supplementary file 2 — Reporting Summary [file 41467_2026_73438_MOESM2_ESM.pdf]

Corresponding author(s): Prof. Jean Marshall

Last updated by author(s): Mar 17, 2026

Reporting Summary

Nature Portfolio wishes to improve the reproducibility of the work that we publish. This form provides structure for consistency and transparency in reporting. For further information on Nature Portfolio policies, see our Editorial Policies and the Editorial Policy Checklist.

Statistics

For all statistical analyses, confirm that the following items are present in the figure legend, table legend, main text, or Methods section.

n/a	Confirmed	
☐	☒	The exact sample size (*n*) for each experimental group/condition, given as a discrete number and unit of measurement
☐	☒	A statement on whether measurements were taken from distinct samples or whether the same sample was measured repeatedly
☐	☒	The statistical test(s) used AND whether they are one- or two-sided<br>*Only common tests should be described solely by name; describe more complex techniques in the Methods section.*
☒	☐	A description of all covariates tested
☐	☒	A description of any assumptions or corrections, such as tests of normality and adjustment for multiple comparisons
☐	☒	A full description of the statistical parameters including central tendency (e.g. means) or other basic estimates (e.g. regression coefficient) AND variation (e.g. standard deviation) or associated estimates of uncertainty (e.g. confidence intervals)
☐	☒	For null hypothesis testing, the test statistic (e.g. *F*, *t*, *r*) with confidence intervals, effect sizes, degrees of freedom and *P* value noted<br>*Give P values as exact values whenever suitable.*
☒	☐	For Bayesian analysis, information on the choice of priors and Markov chain Monte Carlo settings
☒	☐	For hierarchical and complex designs, identification of the appropriate level for tests and full reporting of outcomes
☐	☒	Estimates of effect sizes (e.g. Cohen's *d*, Pearson's *r*), indicating how they were calculated

Our web collection on statistics for biologists contains articles on many of the points above.

Software and code

Policy information about availability of computer code

Data collection	No computer code and algorithm used in this study.
Data analysis	FlowJo™ software version 10.8.1 (BD Biosciences), Prism 6-10, ImageJ (version 2.14.0), and Zen Blue 3.0

For manuscripts utilizing custom algorithms or software that are central to the research but not yet described in published literature, software must be made available to editors and reviewers. We strongly encourage code deposition in a community repository (e.g. GitHub). See the Nature Portfolio guidelines for submitting code & software for further information.

Data

Policy information about availability of data

All manuscripts must include a data availability statement. This statement should provide the following information, where applicable:

- Accession codes, unique identifiers, or web links for publicly available datasets
- A description of any restrictions on data availability
- For clinical datasets or third party data, please ensure that the statement adheres to our policy

The data supporting the findings of this study are available within the article and its Extended Data files. Raw data used to build the figures are available from the corresponding author upon reasonable request.

Research involving human participants, their data, or biological material

Policy information about studies with human participants or human data. See also policy information about sex, gender (identity/presentation), and sexual orientation and race, ethnicity and racism.

Reporting on sex and gender	N/A
Reporting on race, ethnicity, or other socially relevant groupings	N/A
Population characteristics	N/A
Recruitment	N/A
Ethics oversight	N/A

Note that full information on the approval of the study protocol must also be provided in the manuscript.

Field-specific reporting

Please select the one below that is the best fit for your research. If you are not sure, read the appropriate sections before making your selection.

☒ Life sciences ☐ Behavioural & social sciences ☐ Ecological, evolutionary & environmental sciences

For a reference copy of the document with all sections, see nature.com/documents/nr-reporting-summary-flat.pdf

Life sciences study design

All studies must disclose on these points even when the disclosure is negative.

Sample size	Experiments were conducted with 3–14 mice per group, based on prior experience with similar immunological models and observed variability. While no formal statistical method was used to predetermine sample size, this range was sufficient to detect meaningful immune differences, and key findings were confirmed through repeated independent experiments.
Data exclusions	In 2-3 isolated cases lung samples were excluded from flow cytometry analysis due to extremely poor cell recovery following tissue digestion. These exclusion criteria were not pre-established but were applied consistently to avoid unreliable or low-quality data. All remaining samples with sufficient cell yield and viability were included in the analysis.
Replication	All key experimental findings were replicated in multiple independent experiments and the data pooled for statistical purposes (with a sample size 3-6 mice per group per experiment). Consistent trends were observed across experimental repetitions. While, due to limited statistical power, some individual experiments did not reach statistical significance, the directionality of the effects was consistent, supporting the reproducibility of the findings. No findings were identified as non-reproducible.
Randomization	Randomization was not applicable in this study due to the use of genetically distinct mouse strains, such as wild-type and genetically modified mice (e.g., mast cell–deficient or eosinophil-deficient), which were bred and housed separately. Since genotype and experimental group assignment were known in advance and essential for the study design, random allocation was not feasible. However, to reduce potential bias and control for covariates, mice of the same sex and similar age were used across all experimental groups, and animals were handled and processed in parallel under identical experimental conditions.
Blinding	Although full blinding of group allocation was not feasible due to the use of genetically modified mouse strains that required breeding, genotyping, and prior identification, several measures were taken to minimize bias during data collection and analysis. During experimentation, including infection, sample collection, processing, and flow cytometry acquisition, mouse IDs were replaced with experimental or numerical codes to ensure that investigators remained blinded to the genotype or treatment group of each sample. Histological assessments were performed in a blinded manner by two independent investigators to enhance objectivity and reproducibility. Furthermore, downstream assays such as ddPCR, ELISA, and qPCR were conducted by personnel who were unaware of the sample identities and treatment assignments. These measures were implemented to maintain rigor and reduce potential bias throughout the study.

Reporting for specific materials, systems and methods

We require information from authors about some types of materials, experimental systems and methods used in many studies. Here, indicate whether each material, system or method listed is relevant to your study. If you are not sure if a list item applies to your research, read the appropriate section before selecting a response.

Materials & experimental systems

n/a	Involved in the study
☐	☒ Antibodies
☐	☒ Eukaryotic cell lines
☒	☐ Palaeontology and archaeology
☐	☒ Animals and other organisms
☒	☐ Clinical data
☒	☐ Dual use research of concern
☒	☐ Plants

Methods

n/a	Involved in the study
☒	☐ ChIP-seq
☐	☒ Flow cytometry
☒	☐ MRI-based neuroimaging

Antibodies

Antibodies used

Fluorochrome-labeled antibodies against mouse antigens were obtained from BD Biosciences (Mississauga, Canada), BioLegend (San Diego, CA), or Invitrogen (Carlsbad, CA). The antibodies include CD3e, CD4, CD8a, CD11c, CD11b, CD19, CD24, CD25, CD40, CD31, CD44, CD45, CD45R, CD49b, CD80, CD86, CD90.2, CD117, CD127, CD172a, CD206, CD317, Foxp3, F4/80, GATA3, Ly6C, Ly6G, MHCII, MerTK, NK1.1, PD-L1, ROR t, Sca-1, Siglec-F, ST2, and T-bet. Each antibody clone, catalogue number, source, and dilution used for staining are provided in Supplementary Table 1.

Validation

Antibodies used in this study were commercially sourced and validated by the manufacturers. Validation details and supporting information are available on the respective manufacturer websites and referenced in the manuscript where applicable

Animals and other research organisms

Policy information about studies involving animals; ARRIVE guidelines recommended for reporting animal research, and Sex and Gender in Research

Laboratory animals

Mice: C57BL/6J (strain No. 000664), BALB/cJ (strain No. 000651),), B6. Csf2–/– /J (Strain No. 026812), and ΔdblGATA (strain No. 375 005653) mice were obtained from the Jackson Laboratories (Bar Harbor, ME). Cpa3-Cre; Mcl- 376 1fl/fl (Hello Kitty, HK mice) are gift from Drs. S. Galli and M. Tsai (Stanford University, CA).

Wild animals

N/A

Reporting on sex

Since RSV causes more severe disease in males and females tend to be more resilient to infection, we predominantly used male mice in our studies to better model disease pathogenesis.

Field-collected samples

N/A

Ethics oversight

The experimental findings reported in this study are approved by University Committee for Laboratory Animals of Dalhousie University, Halifax, Canada. (Protocol Nos.18-042, 22-028, and 25-023).

Note that full information on the approval of the study protocol must also be provided in the manuscript.

Plants

Seed stocks

N/A

Novel plant genotypes

N/A

Authentication

N/A

Flow Cytometry

Plots

Confirm that:

☒ The axis labels state the marker and fluorochrome used (e.g. CD4-FITC).

☒ The axis scales are clearly visible. Include numbers along axes only for bottom left plot of group (a 'group' is an analysis of identical markers).

☒ All plots are contour plots with outliers or pseudocolor plots.

☐ A numerical value for number of cells or percentage (with statistics) is provided.

Methodology

Sample preparation	Lung Samples: Lung tissues from both infected and uninfected mice were minced into ~1 mm fragments and digested in 4 mg/mL collagenase D and 80 µg/mL DNase I (Roche Diagnostics, Indianapolis, IN) at 37°C for 45 minutes in a shaker incubator. Digested tissue was passed through a wire mesh to obtain single-cell suspensions, which were then centrifuged at 400 × g for 6 minutes at 10°C. Red blood cells were lysed by resuspending the cell pellet in 1 mL of ammonium chloride (ACK) lysis buffer for 3–4 minutes at room temperature, followed by washing with 1× PBS. Lung samples with poor cell recovery after digestion were excluded from flow cytometry analysis.<br>Spleen and Lymph Nodes: Spleens and lymph nodes were mechanically dissociated by passing through a 100 µm mesh to obtain single-cell suspensions. Red blood cells were lysed using 1 mL of ACK lysis buffer for 3–4 minutes at room temperature, followed by washing in 1× PBS.<br>Peritoneal Wash: Peritoneal lavage collection was performed using 3 mL of 0.5% bovine serum albumin (ThermoFisher) and 5 mM EDTA (Invitrogen) in 1× PBS to collect cells from the peritoneal cavity.<br>After collection, all samples were washed and cell viability was assessed. Single-cell suspensions were subsequently used for flow cytometry analysis.
Instrument	Flow cytometry data were acquired using either a BD LSRFortessa™ SORP (BD Biosciences) equipped with four lasers or a BD FACSymphony™ A5 (BD Biosciences) equipped with five lasers.
Software	Flow cytometry data were acquired using BD FACSDiva™ software on the instrument. Data analysis was performed using FlowJo™ software (version 10.8.1, BD Life Sciences). No custom code was used for analysis.
Cell population abundance	Not applicable. No cell sorting was performed, and post-sort fractions were not collected in these experiments.
Gating strategy	Flow cytometry gating strategy for different innate immune cells. Lineage-negative cells were defined as CD45+CD19−CD3−. B cells were identified as CD45+CD19+; T cells as CD45+CD3+; alveolar macrophages as Siglec-Fhigh; neutrophils as Ly6G +CD11b+; eosinophils as Siglec-F+CD11c−; inflammatory monocytes as MHCII− CD11b+Ly6Chigh; dendritic cells (DCs) as MHCII+CD11c+. For innate lymphoid cell type 2 (ILC2). Lineage-negative cells were defined as CD45+CD11c−CD11b−NK1.1 −CD3−CD19− and ILC2 were identified as CD45+CD90.2+ cells, then confirmed for the expression of ST2. For T helper subsets. Cells were first gated on the lymphocyte population, followed by exclusion of dead cells, then CD4+ T cells were selected while excluding CD8+ cells, and CD19−CD4+ cells were then gated to exclude B cells. Within the CD4+CD19− population, T helper cell subsets were identified based on intracellular transcription factor expression (GATA3+, Foxp3+, RORγt+, and T-bet+).

☒ Tick this box to confirm that a figure exemplifying the gating strategy is provided in the Supplementary Information.
