# Supplementary material for: Mast cells support lung eosinophil homeostasis and the acute innate immune response to respiratory syncytial virus
Source: Nat Commun. 2026 May 24;17:6776. doi: 10.1038/s41467-026-73438-w (PMC13385834; doi:10.1038/s41467-026-73438-w)
Supplement: Supplementary file 3 — Transparent Peer Review file [file 41467_2026_73438_MOESM3_ESM.pdf]

nature portfolio

Peer Review File

Mast cells support the acute innate immune response to Respiratory Syncytial Virus and lung eosinophil homeostasis

Corresponding Author: Dr Jean Marshall

This file contains all reviewer reports in order by version, followed by all author rebuttals in order by version.

Version 0:

Reviewer comments:

Reviewer #1

(Remarks to the Author)
This paper shows that mast cells have a protective role in the early phase of the defense against RSV infection in mice. The effect was proposed to be dependent on mast cell-induced eosinophils, but not evidently shown to be dependent on this.
The study is well-written and elegantly performed using a comparison between mast-cell deficient mice and wild type mice from the same strain (C57BL/6), together with reconstitution of mast cells in mast-cell deficient mice. Following up the potential for eosinophils to influence RSV infection, another genetically modified mouse strain was to show this.
The paper adds significant new original data of the field.
The conclusions are supported by the data. The new data that mast cells are important for inhibiting RSV infection are comprehensively shown from different angles. The mast cell-induced increase of eosinophils and the mechanisms suggesting how this appear are solid. However, there is no proof that the mast cell-induced increase of eosinophils was the reason for the reduced RSV infection, which should increase the importance of the paper.
The methodology is clearly described and met the standards in the field and is enough described to reproduce the studies.

Major criticism
To strengthen the influence of mast cells on the viral effect and reciprocal, the effect of RSV on mast cells, the number and localization of mast cells should be evaluated.
The sentence in the introduction “Mast cells have been studied extensively in the context of RSV-associated wheezing or asthma;” is unclear. In which studies do this refer to and what are they showing?
To show the importance for mast cell-induced eosinophils during RSV infection it is understandable that it is complicated to perform mast-deficient experiments ΔdblGATA mice as this strain was on BALB/c background but why was not HK mice reconstituted with Dsf2-/- BMMC not exposed to RSV. Such experiments should have the possibility to verify the importance of the mast cell-induced eosinophils during RSV infection.

Minor criticism
As the study was performed to investigate the role of mast cells on RSV infection, the fourth paragraph in the introduction about eosinophils in the introduction can be omitted. It takes the focus from the initial aim and confuses the reader. It is obviously added because of the findings. The first sentence in this paragraph is also very unclearly described about the association between eosinophils and mast cells.
The second paragraph in the discussion is mainly a further introduction and can be omitted. Any data of relevance for the results should appear in this context of discussing those.

Reviewer #2

(Remarks to the Author)
This is a well-structured and clearly written manuscript that investigates the role of mast cells in early host defense against RSV infection using Cpa3-Cre; Mcl-1^fl/fl (HK) mast cell-deficient mice as well as bone marrow-derived mast cell reconstitution experiments. The authors report that mast cell-deficient mice exhibit increased RSV viral load, greater lung inflammation, increased inflammatory monocyte recruitment and reduced eosinophil recruitment during early RSV infection. They further show that reconstitution with mast cells reduces viral load and restores eosinophil levels. These exciting data show that mast cells, an element of the inflammatory response, may be protective against RSV under certain conditions.

While the data are compelling and the methodology is generally sound, there are some concerns limit the strength of the conclusions.

Major concerns:
1. The proposed mechanism that mast cells restrict RSV replication through type I interferon production is not well supported. Although cultured mast cells produce type I IFNs in response to RSV in vitro (Figure 4), no evidence of decreased RSV-induced type I IFN levels in the HK mice are presented. It is surprising that the authors did not simply measure type I IFN levels in RSV-infected wild-type and HK mice.

2. NK cells and CD8+ cytotoxic T cells are critical mediators of antiviral immunity in RSV infection. The authors survey the effects of the mast cell KO on many different leukocytes in Figure 2, but NK cells and CD8+ T cells are omitted. Indeed, the authors have published that virus-infected mast cells enhance NK cell functions (J Innate Immun 2017). It is therefore conceivable that the mast cell KO mice have increased viral load due to reduced NK cells.

3. Related to concern #2, the authors provide no direct evidence that mast cells themselves prevent replication or kill RSV. NK cells or eosinophils (see below) could also be involved. This could be determined by performing co-culture experiments using primary airway epithelial cells and mast cells to assess the direct effects of mast cells on RSV replication.

4. Since HK mice had a reduction in eosinophils, it is important to show that the loss of mast cells, not eosinophils, is responsible for the increase in RSV viral load. It has previously been shown that eosinophils kill RSV (Rosenberg HF et al. Eosinophils and their interactions with respiratory virus pathogens. Immunol Res 2009). The authors are to be commended for performing experiments in ΔdblGATA mice. While there is no effect on RSV viral load, ΔdblGATA mice lose more weight and have higher inflammatory monocytes than wild type mice. These data suggest the loss of eosinophils, while not sufficient for the increase in RSV viral load, could somehow have contributed to the increase in HK mice.

5. The authors do not provide the age of the mice. This is important because it is unclear to which disease this RSV mouse model is relevant. In general, infants and the elderly are most susceptible RSV (only these populations are approved for RSV vaccines). Experiments in immature or aged mice would be most impactful. Also, the addition of human data would increase impact.

Minor concerns:

1. It appears that the authors have previously published the effects of RSV on mast cell IFN levels (J Allergy Clin Immunol 2015) but this work is not cited.

2. The authors state in their introduction that "Mast cells have been studied extensively in the context of RSV-associated wheezing or asthma..." Can the authors cite a study or two to give the readers an idea about this previous work?

Additional note: An early career researcher co-reviewed this manuscript with one of the established reviewers who provided the listed reports. This is part of the Nature Communications initiative to facilitate training in peer review and to provide appropriate recognition for Early Career Researchers who co-review manuscripts.

Reviewer #3

(Remarks to the Author)
I co-reviewed this manuscript with one of the reviewers who provided the listed reports. This is part of the Nature Communications initiative to facilitate training in peer review and to provide appropriate recognition for Early Career Researchers who co-review manuscripts.

Version 1:

Reviewer comments:

Reviewer #1

(Remarks to the Author)
The authors of have for the revision of the manuscript made extensive experiments which successfully answer the points raised by the reviewers. Furthermore, the text has been majorly improved according to reviewers' comments. A minor comment is that after the revision of text, the statement lines 59-60 “Mast cells have diverse roles in disease pathogenesis and provide 60 distinct responses to different viral pathogens.” now is redundant.

Reviewer #2

(Remarks to the Author)
The authors have responded thoroughly to the major concerns by adding new data and by refining their mechanistic

interpretation of mast cell-derived type 1 IFN mediated antiviral responses during RSV infection. However, a few minor concerns remain.

Minor comments:
1. In the response to point 4, the authors state that “lung eosinophil numbers differ between mast cell–containing and mast cell–deficient mice only after viral load has already diverged.” However, this statement appears to be contradicted by Fig. 6b, which shows reduced lung eosinophil numbers in HK mice at baseline, prior to RSV infection.

2. Interpretation of decreased Tpsb2 expression (Supplementary Fig. 2). If immune cell influx was a major confounding factor for the reduced Tpsb2 expression following RSV infection, changes in bulk qPCR expression would be affected across the dataset (such as decreased IL25 in HK mice which also exhibit increased immune flux compared to WT upon RSV treatment). It would strengthen the conclusion to assess additional mast cell markers (e.g., Kit, Cpa3) to support preserved mast cell abundance, or maybe the authors could remove RNA data for mast cell markers.

3. Labeling and interpretation of IL-25 data (Fig. 3c,d). In Fig. 3c,d, the symbols used for RSV-treated WT and HK mice appear incorrect. In these panels, only RSV-treated mice are shown. The absence of PBS-treated controls limits interpretation, as it is not possible to determine whether the difference was induced by RSV or represent basal expression levels.

4. Support for increased Th cell subsets (Fig. 8a,c). The conclusion that RSV infection increases Th cell subsets is based on comparison between Fig. 8a (RSV-treated) and Fig. 8c (control-treated), which are presented separately and not integrated into a single analysis. Without direct side-by-side comparison, this conclusion is not adequately supported.

Reviewer #3

(Remarks to the Author)
I co-reviewed this manuscript with one of the reviewers who provided the listed reports. This is part of the Nature Communications initiative to facilitate training in peer review and to provide appropriate recognition for Early Career Researchers who co-review manuscripts.

Open Access This Peer Review File is licensed under a Creative Commons Attribution 4.0 International License, which permits use, sharing, adaptation, distribution and reproduction in any medium or format, as long as you give appropriate credit to the original author(s) and the source, provide a link to the Creative Commons license, and indicate if changes were made.
In cases where reviewers are anonymous, credit should be given to 'Anonymous Referee' and the source.
The images or other third party material in this Peer Review File are included in the article’s Creative Commons license, unless indicated otherwise in a credit line to the material. If material is not included in the article’s Creative Commons license and your intended use is not permitted by statutory regulation or exceeds the permitted use, you will need to obtain permission directly from the copyright holder.
To view a copy of this license, visit https://creativecommons.org/licenses/by/4.0/

Response to reviewer's comments

Reviewer #1 comments

This paper shows that mast cells have a protective role in the early phase of the defense against RSV infection in mice. The effect was proposed to be dependent on mast cell-induced eosinophils, but not evidently shown to be dependent on this. The study is well-written and elegantly performed using a comparison between mast-cell deficient mice and wildtype mice from the same strain (C57BL/6), together with reconstitution of mast cells in mast-cell deficient mice. Following up the potential for eosinophils to influence RSV infection, another genetically modified mouse strain was to show this.
The paper adds significant new original data of the field.
The conclusions are supported by the data. The new data that mast cells are important for inhibiting RSV infection are comprehensively shown from different angles. The mast cell-induced increase of eosinophils and the mechanisms suggesting how this appear are solid. However, there is no proof that the mast cell-induced increase of eosinophils was the reason for the reduced RSV infection, which should increase the importance of the paper.
The methodology is clearly described and met the standards in the field and is enough described to reproduce the studies.

Response: We thank the reviewer for their thoughtful and encouraging evaluation of our manuscript. We appreciate the recognition of the rigor of the methodology, and the strength of our mast cell deficiency and reconstitution approaches. We are also grateful for the reviewer's acknowledgement that our study provides significant new insights into the protective role of mast cells during early RSV infection.

Regarding the reviewer's comment on eosinophils, we fully agree that mast cell-dependent eosinophil recruitment is a prominent feature found in our study. To directly test whether eosinophils are required for mast cell–mediated control of RSV, we performed experiments using ΔdblGATA eosinophil-deficient mice, as presented in our submission. As shown in **Fig. 7a-d**, eosinophil-deficient mice exhibited no increase in viral burden during early infection, despite their expected absence in eosinophils. Inflammatory monocyte levels were also unchanged. These results clearly demonstrate that the absence of eosinophils does not impair early RSV control. Therefore, the reduced eosinophil recruitment to the airways in the absence of mast cells is unlikely to account for the increased viral load observed in mast cell–deficient HK mice. This strengthens our conclusion that mast cells exert antiviral effects, directly or indirectly, independent of eosinophils**.**

Additionally, our newly added *in vitro* co-culture data show that soluble mediators released from RSV-infected primary cultured human mast cells, inhibited RSV replication in epithelial cells (**refer to Fig. 4d**). Together, these *in vivo* and *in vitro* findings establish a mast-cell–mediated antiviral impact that is independent of eosinophil recruitment.

We have clarified this interpretation, to ensure the conclusion is clear, in the revised Discussion in paragraph 2.

Major criticism
To strengthen the influence of mast cells on the viral effect and reciprocal, the effect of RSV on mast cells, the number and localization of mast cells should be evaluated.

Response: We thank the reviewer for raising this important point. To determine whether RSV infection alters mast cell abundance in the lung, we first assessed expression of the mast cell–specific gene *Tpsb2*. We observed a reduction in *Tpsb2* transcript levels in RSV-infected WT lungs compared with uninfected WT controls (**Supplementary Fig. 2c**). This decrease may reflect a dilution effect caused by the dramatic influx of non–mast cell immune populations into the lung during acute RSV infection, rather than a true loss of mast cells. Alternatively, it has been demonstrated by others that the M protein of RSV which is expressed and localises to the nucleus in the early hours post-infection can selectively inhibit gene transcription[1] (Li HM et al, 2021). It is possible that this or other viral immune evasion mechanisms that limit host cell transcription are limiting *Tpsb2* expression

To more directly address mast cell abundance at the tissue level, we performed histological quantification. Quantification of mast cells in the tracheal tissues revealed no significant reduction in mast cell density following RSV infection as compared to uninfected WT controls (**Supplementary Fig. 2d**). Consistent with this, histological analysis of the lung parenchyma showed no significant difference in mast cell numbers between infected and uninfected mice (**Supplementary Fig. 2e**).

Collectively, these findings indicate that RSV infection in this model is not associated with a substantial depletion or expansion of mast cell populations in either the tracheal tissue or lung parenchyma. These results have incorporated in the results section.

The sentence in the introduction "Mast cells have been studied extensively in the context of RSV-associated wheezing or asthma;" is unclear. In which studies do this refer to and what are they showing?

Response: Thank you for this helpful comment. Our original sentence was intended to refer to studies that have examined RSV infection primarily in the context of wheezing and asthma-like airway disease, rather than studies directly interrogating mast cell–mediated antiviral responses during RSV infection. We agree that this distinction was not clearly articulated.

To address this, we have revised the statement in the Introduction to clarify that "*While RSV has been extensively studied in relation to wheezing and asthma-associated inflammation* [2, 3, 4]*—conditions in which mast cells are well-established contributors*[5, 6]*—the specific role of mast cells in early antiviral defense and RSV-associated lung pathology remains poorly defined*".

We have therefore revised the sentence in the introduction to clearly reflect this distinction and to better frame the knowledge gap addressed by our study.

To show the importance for mast cell-induced eosinophils during RSV infection it is understandable that it is complicated to perform mast-deficient experiments ΔdblGATA mice as this strain was on BALB/c background but why was not HK mice reconstituted with Dsf2-/- BMMC not exposed to RSV. Such experiments should have the possibility to verify the importance of the mast cell-induced eosinophils during RSV infection.

Response: We thank the reviewer for this thoughtful suggestion and have completed additional experiments to address this issue. As now shown in **Fig. 7e–g**, WT, HK, WT-BMMC–reconstituted HK mice, and *Csf2*$^{-/-}$ BMMC–reconstituted HK mice, were infected with RSV. We were then able to assess the influence of mast cell-derived GM-CSF on eosinophil infiltration into the lung during RSV infection.

WT-BMMC reconstitution of mast cell deficient HK mice was associated with better viral control, following RSV infection compared with that observed in infected HK mice (**Fig. 7e**) Both inflammatory monocyte and eosinophil responses following mast cell reconstitution of HK animals were similar to those observed in wild type mice (**Fig. 7f–g**). Importantly, reconstitution of HK mice with *Csf2-/-* BMMCs produced similar phenotype to that observed following reconstitution with WT- BMMCs. RSV viral loads, eosinophil numbers, and inflammatory monocytes levels were all comparable to those observed in WT-BMMC-reconstituted and WT control mice (Fig. 7e–g). These results indicate that mast-cell regulation of eosinophils and monocytes during early RSV infection does not require mast cell GM-CSF production.

Mast cells are known to regulate eosinophil recruitment, function, and survival through multiple mediators, including CCL5, CCL11, IL-5, and IL-3. Even in the absence of mast cell-derived GM-CSF, mast cells can still produce multiple antivirals and proinflammatory signals and other inflammatory cells can provide a GM-CSF response. Mast cells are a source of multiple cytokines and chemokines, including IL-5 and GM-CSF [7, 8], that promote eosinophil recruitment and survival. Although mast cell–derived GM-CSF contributes to maintaining eosinophil numbers under steady-state conditions, our data indicate that eosinophil infiltration during RSV infection is enhanced by the presence of mast cells but is not solely dependent on GM-CSF. Instead, this important response is likely mediated by a combination of mast cell–derived factors acting together to support eosinophil recruitment and activation and may include some redundancy in mechanisms.

Minor criticism
As the study was performed to investigate the role of mast cells on RSV infection, the fourth paragraph in the introduction about eosinophils in the introduction can be omitted. It takes the focus from the initial aim and confuses the reader. It is obviously added because of the findings. The first sentence in this paragraph is also very unclearly described about the association between eosinophils and mast cells.
The second paragraph in the discussion is mainly a further introduction and can be omitted. Any data of relevance for the results should appear in this context of discussing those.

Response: Thank you for the comments. These points have been taken care in the revised manuscript.

Reviewer #2 comments

This is a well-structured and clearly written manuscript that investigates the role of mast cells in early host defense against RSV infection using Cpa3-Cre; Mcl-1^fl/fl (HK) mast cell-deficient mice as well as bone marrow-derived mast cell reconstitution experiments. The authors report that mast cell-deficient mice exhibit increased RSV viral load, greater lung inflammation, increased inflammatory monocyte recruitment and reduced eosinophil recruitment during early RSV infection. They further show that reconstitution with mast cells reduces viral load and restores eosinophil levels. These exciting data show that mast cells, an element of the inflammatory response, may be protective against RSV under certain conditions. While the data are compelling and the methodology is generally sound, there are some concerns limit the strength of the conclusions.

We thank the reviewer for their thoughtful and encouraging comments on our manuscript. We appreciate the positive comments regarding the clarity, structure, and scientific merit of our manuscript, as well as the recognition of the significance of defining a protective role for mast cells in early RSV infection. We also thank the reviewer for identifying several important points that help strengthen the interpretation and conclusions of our work. In response, we have performed additional analyses, clarified key aspects of the methodology, incorporated revised data, and revised relevant sections of the manuscript accordingly. We address each concern in detail below and explain how the revisions strengthen our study conclusion that mast cells contribute to early antiviral defense and regulation of lung inflammation during RSV infection.

Major concerns:

1. The proposed mechanism that mast cells restrict RSV replication through type I interferon production is not well supported. Although cultured mast cells produce type I IFNs in response to RSV in vitro (Figure 4), no evidence of decreased RSV-induced type I IFN levels in the HK mice are presented. It is surprising that the authors did not simply measure type I IFN levels in RSV-infected wild-type and HK mice.

Response: We thank the reviewer for raising this important point. To directly address whether mast cells restrict RSV replication through type I IFN production *in vivo*, we quantified type I interferon (IFN) responses at the transcriptional level in RSV-infected WT and HK mice at 2 days post-infection. These newly generated data are now included in Supplementary Fig. 3d–e.

Our analyses demonstrate that RSV infection induces comparable type I IFN responses in WT and HK lungs at day 2 post infection. Although *Ifnb1* expression was significantly higher in HK mice, this increase occurred in the absence of significant changes in the expression of multiple IFN-α subtypes (*Ifna1, Ifna2, Ifna14*) and type III IFN (*Ifnl2, Ifnl3*).
Importantly, *Ifnb1* expression showed a strong positive correlation with viral load, indicating that the elevated *Ifnb1* levels observed in HK mice, at the day 2 time point, reflect increased viral replication rather than impaired antiviral signaling or reduced IFN induction. These data therefore argue against a dominant role for mast cells in augmenting bulk lung type I IFN responses during an ongoing RSV infection.

Despite the absence of enhanced whole-lung type I IFN responses in mast cell–sufficient mice at day 2, our *in vitro* data demonstrate that human mast cell–derived soluble factors significantly reduce RSV infection of human airway epithelial cells (BEAS-2B). We interpret the differences in these systems as representing distinct temporal, spatial and mechanistic contexts rather than an inconsistency in our observations**.** *In vivo*, IFN measurements from lung homogenates represent composite signals from multiple immune and non-immune cell populations, particularly during established, acute RSV infection when inflammatory cell influx is substantial. Under these conditions, localized mast cell–dependent impacts may not be detectable in bulk tissue analyses, especially given the rapid and transient kinetics of IFN signaling and the elevated levels of RSV infection in the mast cell-deficient mouse lungs compared with their wild type counterparts.

Notably, mast cells are enriched in the upper airways, which represent the initial site of RSV entry and replication. It therefore remains plausible that mast cell–derived mediators—including IFNs or other antiviral factors—act very early and locally to limit initial epithelial infection, consistent with our *in vitro* observations showing reduced RSV infection of airway epithelial cells exposed to mast cell–conditioned media. These concepts are now included in the discussion

We also noted that the IFN response to RSV extends well beyond the limited set of IFN genes analyzed here. Mast cells are capable of producing multiple IFN-α/β subtypes that may function within spatially restricted microenvironments or at time points not captured in our bulk lung measurements. Moreover, mast cell antiviral responses are not limited to canonical IFN pathways. Mast cells sense RSV through TLR4 engagement by the RSV F protein, triggering a broad cytokine and chemokine program that includes TNF, IL-1β, IL-6, IL-13, IL-5, GM-CSF, IL-10, IL-29, CCL3, CCL2, and CXCL2. These mediators can contribute to epithelial antiviral defense and to early recruitment or modulation of eosinophils, monocytes, and neutrophils. In addition, RSV infection induces the release of host alarmins such as HMGB1, which can further amplify mast cell activation through TLR4 and TLR2 signaling pathways or limit RSV infection.

Consistent with these mechanisms, our *in vitro* experiments show that supernatants from RSV-infected mast cells markedly reduce RSV infection of BEAS-2B airway epithelial cells in the absence of mast cell degranulation. Taken together, our findings support the concept that mast cells limit RSV infection by multiple mechanisms which may include direct and indirect impacts of mast cell derived IFNs, cytokine and chemokine mediated recruitment and activation of antiviral effector cells and by restricting early viral expansion and shaping the local epithelial antiviral environment through soluble mediators downstream of the RSV F protein and/or HMGB1 mediated –TLR4 activation. The balance and impact of these different pathways will likely differ with the site, level and duration of infection and be influenced by a variety of host factors.

These new data have been incorporated into the revised manuscript, and the interpretation has been expanded in the Discussion to clarify the distinction between localized mast cell–mediated antiviral effects and bulk lung IFN responses.

2. NK cells and CD8+ cytotoxic T cells are critical mediators of antiviral immunity in RSV infection. The authors survey the effects of the mast cell KO on many different leukocytes in Figure 2, but NK cells and CD8+ T cells are omitted. Indeed, the authors have published that

virus-infected mast cells enhance NK cell functions (J Innate Immun 2017). It is therefore conceivable that the mast cell KO mice have increased viral load due to reduced NK cells.

Response: We thank the reviewer for this comment. We have analyzed NK cells and $CD8^+$ T cells in the lungs of RSV-infected HK and WT mice and have included these data in **Supplementary Fig. 2a and 2b** (2 and 7 dpi). Our results show no significant differences in the numbers of either NK cells or $CD8^+$ T cells between the two groups at either time point. Additionally, baseline (uninfected) analyses confirmed that NK cell and $CD8^+$ T-cell frequencies are comparable between HK and WT mice (now presented in **Supplementary Fig. 4a**). These findings are now described in the revised Results section of the manuscript.

3. Related to concern #2, the authors provide no direct evidence that mast cells themselves prevent replication or kill RSV. NK cells or eosinophils (see below) could also be involved. This could be determined by performing co-culture experiments using primary airway epithelial cells and mast cells to assess the direct effects of mast cells on RSV replication.

Response: To assess whether mast cells have a direct antiviral effect on RSV we performed co-culture experiments using human airway epithelial BEAS-2B cells and supernatants from human cord blood–derived mast cells (CBMCs). We have shown previously that RSV infection in CBMCs induced cytokines and chemokine responses without degranulation (Al Afif et al. 2015). CBMCs were either mock-treated or infected with RSV, and their supernatants were added to BEAS-2B cultures along with RSV infection. We quantified viral replication by assessing the numbers of GFP^+ RSV-infected epithelial cells. Our results demonstrated that supernatants from RSV-infected CBMCs (UV inactivated) significantly reduced the number of RSV infected (GFP^+) BEAS-2B cells compared with RSV infected BEAS-2B cells that had been treated with control (mock) CBMC supernatants, indicating that mast cell–derived antiviral factors—likely including type I IFN s, but potentially also other mast cell products—can directly control RSV replication in epithelial cells. These data support a model in which mast cells contribute to early viral control through secretion of antiviral mediators rather than via cytotoxic mechanisms. This new dataset has been added to the revised **Fig. 4d** (with corresponding text in the Results section). Notably, our previously reported data on NK cell recruitment and activation by mast cells was entirely in a human cell system and recruitment relied on CXCL8, a chemokine not observed in mice.

4. Since HK mice had a reduction in eosinophils, it is important to show that the loss of mast cells, not eosinophils, is responsible for the increase in RSV viral load. It has previously been shown that eosinophils kill RSV (Rosenberg HF et al. Eosinophils and their interactions with respiratory virus pathogens. Immunol Res 2009). The authors are to be commended for performing experiments in ΔdblGATA mice. While there is no effect on RSV viral load, ΔdblGATA mice lose more weight and have higher inflammatory monocytes than wild type mice. These data suggest the loss of eosinophils, while not sufficient for the increase in RSV viral load, could somehow have contributed to the increase in HK mice.

Response: We thank the reviewer for highlighting the importance of distinguishing mast cell-specific effects from those related to eosinophils. We agree that eosinophils have reported antiviral capabilities, and we were mindful of this when interpreting our findings. To address this point directly, we examined RSV infection outcomes in eosinophil-deficient ΔdblGATA mice.

As shown in **Fig. 7b**, ΔdblGATA mice exhibited no increased viral burden at 2 dpi compared with WT controls, despite displaying enhanced weight loss. This may suggest an important role for eosinophils in regulating the inflammatory response or cytokine milieu. Importantly, and contrary to the reviewer's concern, the levels of inflammatory monocytes at day 2 post infection were not significantly increased in ΔdblGATA mice compared with wild type mice (this is now more clearly denoted in **Fig. 7c**); their numbers remained comparable to WT animals. These findings indicate that eosinophil deficiency alone is insufficient to account for the increased viral burden observed in HK mice at the early time points examined. We fully acknowledge that eosinophils have been shown to exert antiviral functions in other experimental settings and at later stages of RSV infection, including those described in prior studies cited by the reviewer. Furthermore, our results indicate that lung eosinophil numbers differ between mast cell containing and mast cell-deficient mice only after viral load has already diverged, suggesting that reduced eosinophilia is a downstream consequence of altered mast-cell–dependent immune regulation rather than a primary driver of increased viral burden.

Together, these findings support a mast cell–intrinsic antiviral function during the early phase of RSV infection, potentially mediated by mast cell–derived soluble factors and modulation of early monocyte responses, while indicating that eosinophil deficiency does not account for the increased viral burden observed in HK mice under the conditions and time points analyzed. It is important to note that the previously cited study by Rosenberg *et al.* modulated eosinophil numbers through IL-5 overexpression and assessed RSV burden at later time points. Although that study additionally validated findings using ΔdblGATA mice and measured viral load at 5 days post-infection (not at day 2 post-infection), we now recognize that IL-5 exerts broader effects on antiviral immunity beyond eosinophil regulation. Notably, IL-5 can enhance mast cell interferon responses to RSV infection, as recently demonstrated by Barra J *et al.* (JACI, 2025). This clarification has been incorporated into the revised Discussion (paragraph 2 and 4).

5. The authors do not provide the age of the mice. This is important because it is unclear to which disease this RSV mouse model is relevant. In general, infants and the elderly are most susceptible RSV (only these populations are approved for RSV vaccines). Experiments in immature or aged mice would be most impactful. Also, the addition of human data would increase impact.

Response: We appreciate the reviewer's comment regarding the importance of mouse age in RSV disease modeling. In our study, we used 4–6-week-old HK and WT C57BL/6 mice for the primary mechanistic experiments, which is standard, used by others, for dissecting early innate immune responses to RSV. At this age, mice possess immunologically mature mast-cell compartments and stable lung architecture, allowing us to reliably evaluate mast-cell–dependent antiviral mechanisms without the confounding variables of postnatal immune development. Importantly, our mast-cell reconstitution experiments also included groups of substantially older mice**:** HK mice (4-6 weeks) were reconstituted with BMMCs and maintained for an 7–8 weeks to allow mast cell prior to infection. Thus, mast cell reconstituted HK mice, age-matched WT, and HK controls were approximately 12–14 weeks old at the time of RSV challenge, enabling us to assess mast-cell function in both young adult (4–6 weeks) and older (12–14 weeks) mice within this study.

We agree that neonatal and aged mouse models more closely reflect human populations at highest clinical risk. However, RSV infection in neonatal mice presents several limitations for the mechanistic questions addressed here. Neonates have low baseline mast-cell numbers**,** incomplete mast-cell maturation, and rapidly changing stromal and epithelial environments, making it difficult to distinguish mast-cell–intrinsic antiviral pathways from developmental effects. Moreover, neonatal mice exhibit fundamentally different leukocyte recruitment kinetics, TLR signaling thresholds, and interferon responses, all of which would complicate interpretation of how mast cells contribute to early viral control. For these reasons, mechanistic dissection of mast-cell functions—including reconstitution approaches – is more definitive using immunologically mature mice, and thus young adult models remain the field standard for defining cell-specific antiviral pathways.

We agree with the reviewer that studies in neonatal or elderly mice would provide complementary insight into disease susceptibility and clinical severity, and we have added this point to the Discussion to clarify the translational context of our work. Incorporating human data would further enhance the impact of this work, which is beyond the scope of this report.

Minor concerns:

1. It appears that the authors have previously published the effects of RSV on mast cell IFN levels (J Allergy Clin Immunol 2015) but this work is not cited.

This reference has been added.

2. The authors state in their introduction that "Mast cells have been studied extensively in the context of RSV-associated wheezing or asthma..." Can the authors cite a study or two to give the readers an idea about this previous work?
This

We have revised the sentence in the introduction to clearly reflect this distinction and to better frame the knowledge gap addressed by our study along with citations.

Additional note: An early career researcher co-reviewed this manuscript with one of the established reviewers who provided the listed reports. This is part of the Nature Communications initiative to facilitate training in peer review and to provide appropriate recognition for Early Career Researchers who co-review manuscripts.

Reviewer #3 (Remarks to the Author)
We thank reviewer #3 for their comments as well.

I co-reviewed this manuscript with one of the reviewers who provided the listed reports. This is part of the Nature Communications initiative to facilitate training in peer review and to provide appropriate recognition for Early Career Researchers who co-review manuscripts.

References

1. Li, H.M. *et al.* Respiratory Syncytial Virus Matrix Protein-Chromatin Association Is Key to Transcriptional Inhibition in Infected Cells. *Cells* **10** (2021).

2. De Leeuw, E. *et al.* Maternal allergy and neonatal RSV infection synergize via FcR-mediated allergen uptake to promote the development of asthma in early life. *Sci Immunol* **10**, eadz4626 (2025).

3. Kusel, M.M. *et al.* Early-life respiratory viral infections, atopic sensitization, and risk of subsequent development of persistent asthma. *J Allergy Clin Immunol* **119**, 1105-1110 (2007).

4. Sigurs, N., Bjarnason, R., Sigurbergsson, F. & Kjellman, B. Respiratory syncytial virus bronchiolitis in infancy is an important risk factor for asthma and allergy at age 7. *Am J Respir Crit Care Med* **161**, 1501-1507 (2000).

5. Lambrecht, B.N., Ahmed, E. & Hammad, H. The immunology of asthma. *Nat Immunol* **26**, 1233-1245 (2025).

6. Galli, S.J. & Tsai, M. IgE and mast cells in allergic disease. *Nat Med* **18**, 693-704 (2012).

7. Ramirez, G.A. *et al.* Eosinophils from Physiology to Disease: A Comprehensive Review. *Biomed Res Int* **2018**, 9095275 (2018).

8. Pope, S.M. *et al.* Identification of a cooperative mechanism involving interleukin-13 and eotaxin-2 in experimental allergic lung inflammation. *J Biol Chem* **280**, 13952-13961 (2005).

REVIEWERS' COMMENTS

Reviewer #1 (Remarks to the Author):

The authors of have for the revision of the manuscript made extensive experiments which successfully answer the points raised by the reviewers. Furthermore, the text has been majorly improved according to reviewers' comments. A minor comment is that after the revision of text, the statement lines 59-60 "Mast cells have diverse roles in disease pathogenesis and provide 60 distinct responses to different viral pathogens." now is redundant.
Response: We have removed this sentence from the manuscript.

Reviewer #2 (Remarks to the Author):
The authors have responded thoroughly to the major concerns by adding new data and by refining their mechanistic interpretation of mast cell-derived type 1 IFN mediated antiviral responses during RSV infection. However, a few minor concerns remain.

Minor comments:
1. In the response to point 4, the authors state that "lung eosinophil numbers differ between mast cell–containing and mast cell–deficient mice only after viral load has already diverged." However, this statement appears to be contradicted by Fig. 6b, which shows reduced lung eosinophil numbers in HK mice at baseline, prior to RSV infection.
Response: Thank you for pointing out this issue. We have revised our interpretation around this in our manuscript, to ensure consistency with our results showing reduced eosinophil numbers in HK mice prior to RSV infection.

2. Interpretation of decreased Tpsb2 expression (Supplementary Fig. 2). If immune cell influx was a major confounding factor for the reduced Tpsb2 expression following RSV infection, changes in bulk qPCR expression would be affected across the dataset (such as decreased IL25 in HK mice which also exhibit increased immune flux compared to WT upon RSV treatment). It would strengthen the conclusion to assess additional mast cell markers (e.g., Kit, Cpa3) to support preserved mast cell abundance, or maybe the authors could remove RNA data for mast cell markers.
Response: We agree with the reviewer. We have therefore removed the RNA expression data (*Tpsb2*) for mast cell markers from Supplementary Fig 2. c-Kit is not exclusively associated with mast cells and is also expressed by other immune cells, including T cells, which makes it less suitable for assessing mast cell abundance in this context. Although Cpa3 is considered a mast cell–specific marker, we were uncertain whether its expression could be affected under these experimental conditions. Therefore, to avoid potential misinterpretation, we have removed the RNA data for mast cell markers from Supplementary Fig. 2. We now provide histological counting as a more reliable direct measure of tissue mast cells in our revised submission. Mast cells were counted in tracheal tissue and lung parenchyma of uninfected and RSV-infected WT mice. These data are provided in **Supplementary Fig. 2**.

3. Labeling and interpretation of IL-25 data (Fig. 3c,d). In Fig. 3c,d, the symbols used for RSV-treated WT and HK mice appear incorrect. In these panels, only RSV-treated mice are shown. The absence of PBS-treated controls limits interpretation, as it is not possible to determine whether the difference was induced by RSV or represent basal expression levels.
Response: We also examined the baseline expression of these genes in WT and HK mice. No differences were observed in the basal expression levels of *Il25, Il33, Tslp, or Muc5b* between the two groups. Data were analyzed by two-sided Mann-Whitney U test. These data have now been included in

Supplementary Fig. 2e to clarify that the differences observed in **Fig. 3c,d** are associated with RSV infection rather than baseline expression differences. We agree with the reviewer that the symbols were somewhat confusing when PBS-treated WT and HK mice were included. To address this issue, we have revised the figure by assigning different color codes to the uninfected mice in panels where they are presented alongside RSV-infected mice.

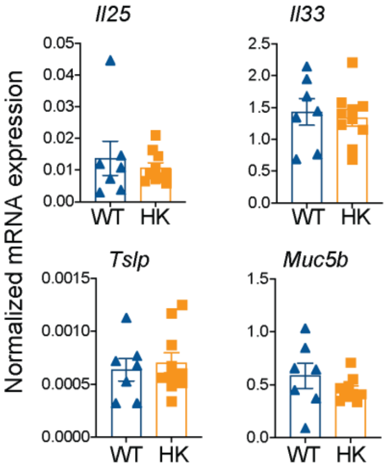

4. Support for increased Th cell subsets (Fig. 8a,c). The conclusion that RSV infection increases Th cell subsets is based on comparison between Fig. 8a (RSV-treated) and Fig. 8c (control-treated), which are presented separately and not integrated into a single analysis. Without direct side-by-side comparison, this conclusion is not adequately supported.

Thank you for this comment, which has encouraged us to improve the presentation of these data. We have revised the presentation of these data. In the updated **Figure. 8c**, baseline control data are now shown alongside the Th cell subset data from **Figure. 8a** within a single integrated panel, allowing direct side-by-side comparison between control and RSV-infected groups. Statistical analysis was performed using one-way ANOVA with Holm–Sidak's multiple-comparison test. This analysis shows that RSV infection does not significantly increase Th1 cell frequencies but is associated with changes in other $CD4^+$ T helper cell subsets, including Th2, Th17, and $Foxp3^+$ cells. This observation is consistent with previous reports (PMID: 21133663, 20404812) indicating that RSV infection can induce type-2-skewed immune responses in the lung. The revised Fig. 8c and the corresponding text in the manuscript have been updated accordingly.

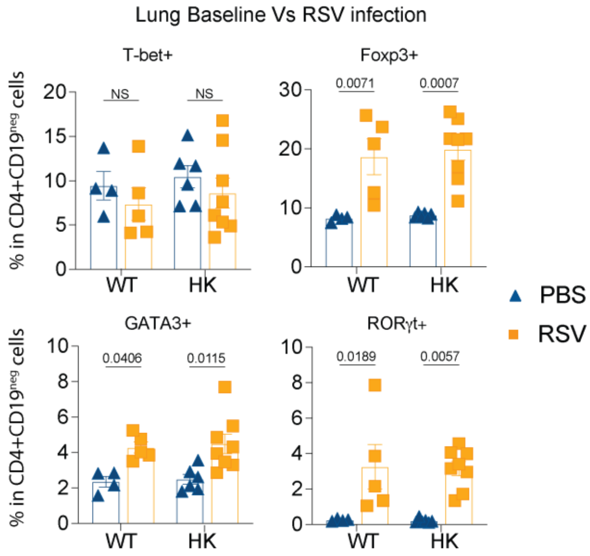

Lung Baseline Vs RSV infection
T-bet+
Foxp3+
GATA3+
RORγt+
% in CD4+CD19neg cells
NS
0.0071
0.0007
0.0406
0.0115
0.0189
0.0057
WT
HK
PBS
RSV
